# Vanadium Carbide Quantum Dots Exert Efficient Anti‐Inflammatory Effects in Lipopolysaccharide‐Induced BV2 Microglia and Mice

**DOI:** 10.1002/smsc.202300334

**Published:** 2024-09-10

**Authors:** Zhijun He, Qiqi Yang, Xiaoqian Li, Zi Wang, Shengwu Wen, Ming‐Jie Dong, Weiyun Zhang, Youcong Gong, Zijia Zhou, Qiong Liu, Haifeng Dong

**Affiliations:** ^1^ Shenzhen Key Laboratory of Marine Biotechnology and Ecology, College of Life Sciences and Oceanography Shenzhen University Shenzhen Guangdong 518055 China; ^2^ School of Modern Industry for Selenium Science and Engineering Wuhan Polytechnic University Wuhan 430023 China; ^3^ Marshall Laboratory of Biomedical Engineering, Precision Medicine and Health Research Institute, Shenzhen Key Laboratory for Nano‐Biosensing Technology, Guangdong Key Laboratory of Biomedical Measurements and Ultrasound Imaging, School of Biomedical Engineering, Shenzhen University Medical School Shenzhen University Shenzhen Guangdong 518060 China; ^4^ Beijing Key Laboratory for Bioengineering and Sensing Technology Department of Chemistry & Biological Engineering University of Science and Technology Beijing Beijing 100083 China; ^5^ Guangdong Laboratory of Artificial Intelligence and Digital Economy (SZ) Shenzhen Guangdong 518107 China; ^6^ Shenzhen‐Hong Kong Institute of Brain Science‐Shenzhen Fundamental Research Institutions Shenzhen Guangdong 518055 China

**Keywords:** anti‐neuroinflammations, blood–brain barrier permeabilities, endoplasmic reticulum stress, oxidative stress, vanadium carbide quantum dots

## Abstract

The regulation of glial cell activation is a critical step for the treatment or prevention of neuroinflammation‐based brain diseases. However, the development of therapeutic drugs that pass the blood–brain barrier (BBB) and inhibit the glia cell activation remains a significant challenge. Herein, an ultrasmall 2D vanadium carbide quantum dots (V_2_C QDs) that are capable of crossing the BBB are prepared, and the admirable anti‐neuroinflammatory effects are presented. The prepared 2D V_2_C QDs with an average size of 2.54 nm show good hydrophilicity, physiological stability, and effective BBB‐crossing ability. The biological effect of V_2_C QDs on inflammatory reactions demonstrates fascinating results in preventing the impairment of learning and memory in BALB/c mice stimulated by lipopolysaccharide. Investigation of molecular mechanism reveals that V_2_C QDs not only inhibit the toll‐like receptor 4/myeloid differentiation factor 88‐mediated nuclear factor kappa B and mitogen‐activated protein kinase pathways, but also prevent eukaryotic translation initiation factor 2α/activating transcription factor 4/C/EBP homologous protein‐signaling pathway and reduce oxidative stress via activating the NF‐E2‐related factor‐2/heme oxygenase‐1‐signaling pathway, leading to greatly inhibited activation of microglia and astrocytes and weakened production of inflammatory cytokines. In summary, V_2_C QDs exert potent anti‐inflammatory effects through multiple pathways, thus offer great potential for the treatment of neurodegenerative diseases.

## Graphical abstract

Development drugs that cross the blood–brain barrier (BBB) and inhibit neuroinflammation is crucial for treating encephalopathy such as Alzheimer's disease. Ultrasmall 2D vanadium carbide quantum dots (V2C QDs) were investigated for this purpose and demonstrated good hydrophilicity, physiological stability and effective BBB‐crossing ability. By inhibiting neuroinflammation, V2C QDs significantly improve cognitive deficit in BALB/c mice stimulated by lipopolysaccharide.

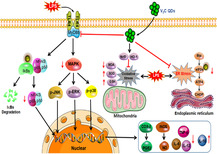

## 1 Introduction

Neuroinflammation has been widely reported as a critical factor leading to cognitive impairment in neurodegenerative diseases (NDs), such as Alzheimer's disease (AD), ischemic stroke, and Parkinson's diseases.^[^ ^1^, ^2^, ^3^, ^4^, ^5^ ^]^ Hyperactivation of microglia and astrocytes is one of the most frequent events in the pathological processes underlying these conditions.^[^ ^6^, ^7^ ^]^ Aberrant inflammatory responses harm to the central nervous system (CNS) via increased levels of various pro‐inflammatory mediators, such as reactive oxygen species (ROS) and cytokines.^[^ ^8^, ^9^, ^10^ ^]^ Accordingly, inhibition of neuroinflammatory reactions has the potential to prevent the development of NDs. Thus, both versatile delivery and efficient anti‐inflammatory approach contribute to the good therapeutic performance against NDs. The efficient and noninvasive delivery of therapeutic drugs to the brain is still an unmet need to the treatment of NDs. One major obstacle to brain delivery is the physiologically yet dynamically functioning blood–brain barrier (BBB).^[^ ^11^ ^]^ The BBB has an essential protection to the CNS; however, it also limits the crossing of drug and thereby makes the treatment of CNS disorders complicated. Thus, it is imperative to develop therapeutic drugs to efficiently pass the BBB.^[^ ^12^ ^]^

Neuroinflammatory reactions are generally related to multiple complicated signal pathways. Lipopolysaccharide (LPS) is widely explored to trigger inflammatory reaction in living cell and in vivo for anti‐inflammatory study.^[^ ^13^ ^]^ It is a component of the membrane of gram‐negative bacteria and is an effective stimulator of glial cells and triggers inflammatory reactions in microglia by recognizing and binding to toll‐like receptor 4 (TLR4).^[^ ^8^ ^]^ After binding to LPS, TLR4 recruits an intracellular adaptor protein, myeloid differentiation factor 88 (MyD88), which subsequently initiates downstream mitogen‐activated protein kinase (MAPK) and nuclear factor kappa B (NF‐κB)‐signaling pathways. Activated MAPK and NF‐κB further induces the transcription of pro‐inflammatory molecules such as interleukin (IL)‐1β, IL‐6, IL‐12, tumor necrosis factor (TNF)‐α, inducible nitric oxide synthase (iNOS) and cyclooxygenase(COX)‐2, and reactive oxygen and nitrogen species.^[^ ^14^, ^15^ ^]^ Accordingly, targeting the regulation of MAPK and NF‐κB‐signaling pathways activation could be a beneficial approach for treating inflammatory diseases.

Peripherally, systemic LPS treatment promotes oxidative stress in the brain. Previous studies have indicated that ROS generation is accelerated by LPS, which increases the malondialdehyde (MDA) and NO, and reduces the levels of the antioxidants superoxide dismutase (SOD) and glutathione (GSH) in the brain.^[^ ^16^ ^]^ Heme oxygenase‐1 (HO‐1), a rate‐limiting catabolic enzyme of heme, drives an adaptive survival reaction that protects cells from oxidative stress and is regulated by NF‐E2‐related factor‐2 (Nrf2).^[^ ^17^ ^]^ As a regulator of the antioxidant system, Nrf2 upregulates HO‐1 expression to reduce oxidative stress. In addition, activated Nrf2/HO‐1 also can inhibit the inflammatory response by regulating the generation of proinflammatory mediators such as NO and cytokines. Thus, targeting the Nrf2/HO‐1‐signaling pathway activation is attracting increasing interest as a possible therapeutic method for reducing oxidative stress and inflammation.

Accumulating evidence indicates that LPS can also cause endoplasmic reticulum (ER) stress, which further contributes to neuronal injury.^[^ ^18^ ^]^ ER stress is a coordinated adaptive process initiated by three ER stress‐related sensors, namely the inositol‐requiring enzyme‐1, protein kinase receptor‐like ER kinase (PERK), and activating transcription factor 6 (ATF6).^[^ ^19^ ^]^ Molecular chaperone glucose‐regulated protein 78 (Bip) generally binds to misfolded proteins and works as a major regulator of ER function. The binding of abnormal proteins to Bip in the cytoplasm results in the dissociation of Bip from the ER‐localized transmembrane sensors; this dissociation then subsequently triggers ER stress by increasing the activity of eukaryotic translation initiation factor 2α (eIF2α) and the expression of ATF4 and C/EBP homologous protein (CHOP). Importantly, recent research has demonstrated that ER stress is also involved in the pathogenesis of inflammation.^[^ ^20^, ^21^ ^]^ Thus, inhibition of ER stress represents another potential approach to suppressing neuroinflammation.

The development of anti‐inflammatory in multiple pathways is potent but has never explored. To the best of our knowledge, there is scarcely an anti‐inflammatory approach that simultaneously modulates the TLR4/MyD88‐mediated NF‐κB and MAPKs pathways, the eIF2α/ATF4/CHOP‐signaling pathway, and oxidative‐stress‐related Nrf2/HO‐1‐signaling pathway. Vanadium carbide nanostructures like V_2_C QDs, V_2_C MXene nanoenzyme, and V_4_C_3_ nanosheets have been regarded as a thriving class of multifunctional materials in numerous potential biomedical applications including theranostics, biosensing, bioimaging, and antibacterial, attributed to their physicochemical diversity and tailorability.^[^ ^22^, ^23^, ^24^ ^]^ For instance, 2D V_2_C MXene was used as a guiding nanozyme to implement anti‐oxidative behaviors for ROS elimination under pathophysiological conditions. V_2_C QDs exhibited an intense photothermal effect at the near Infrared ray II (NIR‐II) biowindow for photothermal therapy and good capability for fluorescent imaging, photoacoustic imaging, and magnetic resonance imaging and was used for anticancer treatment. Herein, we fabricated an ultrasmall multifunctional 2D vanadium carbide quantum dots (V_2_C QDs) for multitarget anti‐inflammatory. The 2D V_2_C QDs displayed good hydrophilicity, physiological stability, and versatile BBB crossing ability. After V_2_C QDs were intraperitoneally injected to LPS‐induced neuroinflammation mouse model, administration of V_2_C QDs greatly reduced neuroinflammation via modulating MAPKs and NF‐κB‐signaling pathways, ameliorated ER stress via inhibiting eIF2α/ATF4/CHOP‐signaling pathway, and suppressed oxidative stress via activating Nrf2/HO‐1‐signaling pathway. Consequently, the V_2_C QDs effectively attenuated cognitive deficits in LPS‐induced mice, and it is emerging as a therapeutic candidate for treatment of inflammation‐related NDs.

## 2 Experimental Section

### 2.1 Materials and Reagents

V_2_AlC powder was obtained from Jilin 11 Technology Co., Ltd. (Jilin, China). Tetrapropylammonium hydroxide (TPAOH, 25% in water) was purchased from Macklin Biochemical Technology Co., Ltd. (Shanghai, China). Oligonucleotides were ordered from Bei Jing Hippo Bio‐Technology Co., Ltd. (Beijing, China) and purified using high‐performance liquid chromatography. The sequences were as follows: 1) Target miRNA‐34a DNA: 5′‐ TGT TGG TCG ATT CTG TGA CGG T‐3′. 2) Detection probe: 5′‐Cy5‐TCGATTCTGTGATTTTACCGTCACAGAAT (BHQ‐2) CGACCA ACA‐3′.

LPS, paraformaldehyde, and Griess reagent were obtained from Sigma‐Aldrich. (St. Louis, MO, USA). Fetal bovine serum (FBS), phosphate‐buffered saline (PBS), Dulbecco's modified Eagle's medium (DMEM) high glucose medium, and trypsin were purchased from Gibco (Grand Island, NY, USA). Murine prostaglandin E_2_(PGE_2_), TNF‐α, IL‐6, IL‐10, and IL‐12 platinum enzyme‐linked immunosorbent assay (ELISA) kits with pre‐coated plates were purchased from Neobioscience Technology Co., Ltd. (Shenzhen, China). All reagents used in this study were of analytical grade.

### 2.2 Preparation and Characterization of V_2_C Sheets and V_2_C QDs

V_2_AlC (2 g) powder was added slowly to 40 mL 50% hydrofluoric acid (HF) solution and the mixture was vigorously stirred for 2 days at room temperature (RT). The etching process can break of V—Al bonds in V_2_AlC to eliminate Al. Then, the resulting V_2_C powder was repeatedly washed with deionized water and dispersed in TPAOH (25 wt% aqueous solution) and the ultrasound‐assisted exfoliation of the multilayer V_2_C in TPAOH (25% in water) was carried out overnight. The V_2_C nanosheets were collected by washing with water and ethanol to remove the free TPAOH and centrifugation at 11 000 rpm for 15 min; they were then freeze‐dried to obtain V_2_C nanosheet.^[^ ^24^, ^25^ ^]^

V_2_C QDs were synthesized according to our previous report with some modification.^[^ ^24^ ^]^ V_2_C nanosheets (5 mg) and 30 mL deionized water were added to a poly(tetrafluoroethylene) (Teflon) lined, stainless‐steel autoclave (50 mL), and the pH of the solution was adjusted to 9.0 by adding ammonium hydroxide. Then, the autoclave was maintained at a temperature of 160 °C for 12 h, the supernatant was dialyzed for 3 d to produce purified V_2_C QDs.

The nanomaterials were analyzed by high‐resolution scanning electron microscopy (SEM, APREO S, Thermo Scientific) to characterize their morphology and elemental analysis. The size and lattice parameter of the V_2_C QDs were detected using a field‐emission transmission electron microscope (TEM, JEM‐F200, Tokyo, Japan). The valence state of vanadium was analyzed using X‐ray photoelectron spectroscopy (XPS, ESCALAB Xi, Thermo Scientific).

### 2.3 Animals and V_2_C QDs Administration

Adult male BALB/c mice (8 weeks old, weighing 20–22 g) were purchased from the Experimental Animal Center (Guangzhou, China). All mice were housed in the Animal Center of Shenzhen University under conditions of controlled temperature (22 ± 1 °C) and humidity (50% ± 10%). The mice had free access to standard food pellets and pure water under a controlled 12 h light/dark cycle (light on between 08:00 and 20:00). Animal welfare and all experimental procedures with animals were carried out in strict accordance with the guidelines of the Institutional Animal Care and Use Committee of the Institute for Nutritional Sciences in China, and the animal protocol was approved by the Regional Ethical Committee for Animal Experimentation at Shenzhen University (approval no. 20140615‐002).

After 1 week of adaption in the animal care facility, 120 mice were randomly divided into four experimental groups (*n* = 30 mice per group) as follows: control group (Ctr), LPS group (LPS), LPS + V_2_C QDs (5 mg kg^−1^) group, and LPS + V_2_C QDs (10 mg kg^−1^) group. V_2_C QDs were dissolved in saline and administered to the mice via intraperitoneal injection at doses of 5 or 10 mg kg^−1^ daily for 9 consecutive days. On day 9, all mice except for those in the control group were intraperitoneally injected with 5 mg kg^−1^ LPS. Behavioral tests were carried out 24 h after the last LPS injection (Figure 3A).

### 2.4 Behavioral Evaluation

Cognitive and behavioral tests were conducted on the animals before they were sacrificed. The Y‐maze test, elevated plus maze, and open field test were performed to assess the effect of V_2_C QDs on anxiety levels, locomotion, exploration, memory, and learning ability in LPS‐induced mice. All behavioral tests were carried out in quiet surroundings, as per our previous publications.^[^ ^26^ ^]^

For the Y‐maze test, the Y‐maze device consisted of three identical arms (designated A, B, and C), each measuring 40 cm long × 20 cm high × 10 cm wide (Figure 3B). Each mouse was placed at the distal end of the same arm and allowed to freely explore the maze for 5 min. Spontaneous alternations were defined as consecutive entries into three different arms and calculated as follows: number of successful alternations/(total number of arm entries −2).

For the elevated plus maze task, the plus sign‐shaped apparatus consisted of two opposite closed arms (50 cm long × 5 cm wide × 15 cm tall) and two opposite open arms (50 cm length × 5 cm wide) at a height of 40 cm (Figure 3E). Each mouse was put in the central area of the maze facing the same open arm and allowed enter all arms freely for 5 min. The time spent in the open arms and number of open‐arm entries were recorded during the 5 min.

For the open field test, the apparatus was a plastic open field (100 cm long × 100 cm wide × 40 cm tall) with its floor divided into 25 equal‐sized squares. Each mouse was placed at the center of the floor and allowed to freely explore the open apparatus for 5 min after a 1 min of acclimatization. The number of grids crossed, as well as rearing and fecal behavior, were monitored in the open field. Between trials, the apparatus was cleaned with 75% ethanol to avoid odor cues.

### 2.5 Tissue Preparation and Brain Protein Extraction

After completing the behavioral tests, the mice were deeply anesthetized with isoflurane and then euthanized. The brain tissue of the mice was harvested immediately. Six hemispheres from each group were preserved in 4% paraformaldehyde for 48 h, embedded in paraffin, and cut into 5 μm sections for further histological analysis. Hippocampus tissue was homogenized in radioimmunoprecipitation assay lysis buffer supplemented with phenylmethylsulfonyl fluoride, protease inhibitors, and phosphatase inhibitor. Then, the protein samples were centrifuged at 13 000 × g for 30 min at 4 °C, and the supernatants were collected for further biochemical analysis.

### 2.6 Western Blot Analysis

The concentrations of total protein extracts from brain tissue and cultured BV2 cells were determined using the bicinchoninic acid (BCA) protein assay kit (Thermo Fisher, USA). Equal amounts of total protein (30 μg) were separated on 10% or 12% sodium dodecyl sulfate–polyacrylamide (PAGE) gels and then transferred to polyvinylidenedifluoride membranes (Millipore, USA) for immunoblotting. Next, membranes were blocked with 5% bovine serum albumin (BSA) at RT for 3 h and probed with different primary antibodies (Table S1, Supporting Information) at 4 °C overnight. On the second day, the membranes were rinsed with Tris‐buffered saline containing 0.5% Tween 20 (TBST) and then incubated with horseradish‐peroxidase‐conjugated secondary antibodies for 2 h at RT. After three washes with TBST, proteins were detected using enhanced chemiluminescent detection reagent. Band densities were analyzed using ImageJ software (NIH, Baltimore, MD, USA).

### 2.7 ELISA

For ELISA assays, serum, hippocampus tissues, and cell culture medium were collected after treatment. The concentrations of PGE_2_, TNF‐α, IL‐6, IL‐10, and IL‐12 were measured using commercially available ELISA assays according to the manufacturer's instructions and quantified using a microplate reader at 450 nm.

### 2.8 Nitric Oxide Assay

Supernatants were collected from BV2 cells in the presence or absence of LPS. NO concentrations in the supernatants were then determined using a NO assay kit according to the manufacturer's instructions (Beyotime, Shanghai, China).

### 2.9 Immunofluorescence Staining

Immunofluorescence staining was conducted as described previously.^[^ ^26^ ^]^ For the immunofluorescent staining of brain sections, 5 μM paraffin sections of brain tissue were deparaffinized and rehydrated, followed by antigen retrieval in citric acid buffer. All sections were permeabilized with 0.3% Triton X‐100 for 15 min and then blocked with 5% BSA for 2 h. Next, the sections were incubated with primary antibodies at 4 °C overnight, rinsed with PBS three times, and incubated with the respective fluorescent secondary antibody. Counterstaining was carried out using 4,6‐diamidino‐2‐phenylindole (DAPI). After three rinses in PBS, the immunolabeled slides were mounted with a mounting media on coverslips.

For the immunofluorescent staining of cells, the BV2 cells were fixed with 4% paraformaldehyde and permeabilized in 0.1% Triton X‐100. Subsequently, the cells were blocked with 5% BSA and incubated overnight with primary antibodies at 4 °C. After several rinses in PBS, the cells were stained with DAPI in the dark for 10 min and then washed with PBS. The cells were observed and imaged using an laser scanning microscopes (LSM 880, Zeiss Germany) Non‐linear optical (NLO)laser scanning system was equipped with the confocal microscope. Fluorescent images were quantified using Image J software 2.0.

### 2.10 Cell Culture and Treatments

Immortalized mouse BV2 cells were purchased from the Institute of Biochemistry and Cell Biology, Chinese Academy of Sciences, and cultured at 37 °C, 5% with CO_2_ in a mixed medium containing 89% DMEM and 10% heat‐inactivated FBS supplemented with 1% penicillin/streptomycin. After reaching 70% confluency, the BV2 cells were pretreated with 2, 5, or 10 μg mL^−1^ V_2_C QDs for 2 h and subsequently stimulated with 0.5 μg mL^−1^ LPS for an additional 22 h.

### 2.11 Cell Viability Assay

The BV2 cells and bEnd.3 cells were cultured in 96‐well plates separately, and viability was assessed using a cell counting kit‐8 assay (CCK‐8; Beyotime, Shanghai, China) according to the manufacturer's instructions. Briefly, after incubating with a series of different concentrations of V_2_C QDs, 10 μL of CCK‐8 working solution was added to each well, and the cells were maintained in a 95% humidified atmosphere for 1.5 h at 37 °C. Absorbance at 450 nm was detected using an automatic microplate reader (Thermo Fisher Scientific, Hudson, NH, USA).

### 2.12 TEM

Cells were fixed with 2.5% glutaraldehyde and 1% osmium tetroxide for 2 h at 4 °C. After washing with distilled water for three times, samples were then dehydrated in graded ethanol solutions and embedded in epoxy resin. Next, the samples were cut into ultrathin sections by a Leica ultramicrotome, stained with 2% uranyl acetate and 2% lead citrate. Images were obtained and analyzed by a TEM (FEI Tecnai G2 Spirit).

### 2.13 Inductively Coupled Plasma Mass Spectrometry Analysis

Mouse brain tissue was digested with 8% pure nitric acid at 200 °C using the microwave digestion system (CEM‐Mars6) for 3 h. After digestion, each sample was collected and then diluted with deionized water to reach 20 mL. The levels of vanadium (V) in the mouse brain were detected using inductively coupled plasma mass spectrometry (ICP–MS) (NexIon 300D, PerkinElmer, USA).

### 2.14 In Vitro BBB Transwell Assay

The BBB penetration capability of V_2_C QDs was evaluated with an in vitro BBB model established by bEnd.3 cell line. The bEnd.3 cells (1.0 × 10^5^ cells/well) were seeded into the polycarbonate 24‐well transwell membranes with mean pore size of 1.0 μm and surface area of 0.33 cm^2^ (FALCON cell culture inserts, Becton Dickinson Labware, USA), cultured for several days. The trans‐endothelial electrical resistance (TEER) of the BBB model was monitored by Millicell ERS (electrical resistance system, Millipore, USA). When the TEER exceeded 200 Ω cm^2^, it is indicated that the integrity of the BBB model in vitro model was achieved. Then, the BV2 cells were plated on the bottom chambers. The serum‐free medium and 15 μg mL^−1^ V_2_C QDs were added into the upper chambers. At 1, 2, 4, and 8 h of incubation, the fluorescence intensity of V_2_C QDs in the lower chambers was detected with a Cytation 5 reader (BioTek Instruments Inc.). The amount of V_2_C QDs across BBB was calculated on the basis of standard curve. The drug permeability (%) of V_2_C QDs was calculated as the accumulated amount of V_2_C QDs passing through the monolayer divided by the total amount of V_2_C QDs.

### 2.15 Characterization of In Vitro Detection of miRNA‐34a

DNA with the same sequence as the target miRNA‐34a was used to evaluate the feasibility of the detection probe for miRNA‐34a analysis. First, the detection probe was annealed by heating it at 95 °C for 4 min and then cooling down it to 25 °C at a rate of 1 °C min^−1^. The reactions (10 μL) were prepared by mixing the target and probe in 1×PBS buffer to a final concentration of 1 μM. The mixtures were incubated at 37 °C for 2 h. Then, the assembly of miRNA‐34a and probe was characterized using 10% PAGE electrophoresis. Target, probe, and product mixture (5 μL/1 μM) were mixed with 1 μL 6 × loading buffer. After being run at 90 V for 30 min, the gel was stained with GelRed and imaged using the GELDOC Go Imaging System (USA, BIO‐RAD).

For fluorescence analysis, the probe sequences were first prepared in advance by annealing as mentioned earlier. Then, the probe sequences (200 nM) were mixed with solutions containing different concentrations of target (0, 2, 10, 50, 100, and 200 nM) in 1 × PBS buffer (100 μL) and incubated at 37 °C for 2 h. The solutions were analyzed using a fluorescence spectrophotometer FS5 (UK, Edinburgh Instruments) at 632 nm. All experimental data were analyzed using Origin 2021.

### 2.16 In Vivo Fluorescence Imaging

Different groups of non‐treated and LPS‐stimulated mice were injected with 300 μL PBS, V_2_C QDs (10 mg kg^−1^), probe (20 nmol kg^−1^), or V_2_C QDs@probe (10 mg kg^−1^ V_2_C QDs and 20 nmol kg^−1^ probe). In vivo fluorescence imaging was performed using the IVIS Spectrum imaging system (USA, Perkin Elmer).

### 2.17 Measurement of Intracellular ROS Level

Intracellular ROS generation was measured using a dichlorofluorescein diacetate (DCFH–DA) fluorescence probe (Sigma‐Aldrich). In brief, BV2 cells were treated with LPS and V_2_C QDs, and the neurobasal medium was removed from the cells. Then, 10 μM DCFH–DA in serum‐free medium was added to each well, and the cells were incubated for 1 h at 37 °C. DCF fluorescence intensity was determined at 485 nm excitation and 535 nm emission using a fluorescence plate reader (Molecular Devices, Sunnyvale, CA).

### 2.18 MDA Content, GSH Level, and SOD Activity Assays

Supernatants from tissue samples and BV2 cells were collected, and total protein concentration was detected using the BCA assay kit. MDA concentration was measured using an MDA assay kit (Beyotime, China) using the thiobarbituric‐acid‐reactive substances method. GSH level and SOD enzyme activity were quantified using assay kits according to the manufacturer's instructions (Jiancheng Bioengineering Institute, Nanjing, China). The results were normalized according to the total protein concentration in each sample.

### 2.19 Blood Compatibility Assay

Blood compatibility was determined according to the method of Mariya et al.^[^ ^27^ ^]^ with slight modifications. Blood samples from adult mice were collected into tubes and held for 2 h at RT, followed by centrifugation at 2000 rpm for continuous 15 min. Then, the supernatant was discarded and red blood cells was washed with PBS for three times, and diluted to 2% (v/v) with PBS for later use. An amount of 500 μL V_2_C QDs was placed into the centrifuge tube and then incubated for continuous 30 min. Subsequently, the same volume of above red blood cell suspension was added into the tube and incubated in a water bath for another 30 min at 37 °C. The positive control consisted of blood with water, while blood with PBS served as the negative control. After incubation, the blend was centrifuged at 2000 rpm for 15 min and the supernatant was collected. An amount of 200 μL supernatant was placed in a 96‐well plate and the optical density (OD) was detected at 540 nm wavelength with a microplate reader. The level of hemolysis was finally calculated in accordance with the following equation:

(1) $$\text{Hemolysis rate}=\;\frac{{\text{OD}}_{\text{sample}}-{\text{OD}}_{\text{negative control}}}{{\text{OD}}_{\text{positive control}}-{\text{OD}}_{\text{negative control}}}\;\times 100\%$$

### 2.20 Endpoint Chromogenic Limulus Amebocyte Lysate Assay

In the endpoint chromogenic limulus amebocyte lysate (LAL) assay (Chromogenic LAL Endotoxin Assay Kit; Beyotime, Shanghai, China), measurements were performed according to the manufacturer's instructions. Briefly, the V_2_C QDs and endotoxin standards were added in lysate and mixed gently followed by incubation at 37 °C for 25 min. Then, the chromogenic substrate was added subsequently and incubated at 37 °C for 6 min. The absorbance of each sample was detected at 545 nm with a Cytation 5 reader (BioTek Instruments Inc.). For each sample, the dilution ratio was determined and blank control and inhibition/enhancement controls (ICEs) samples were prepared,^[^ ^28^ ^]^ by spiking a known concentration of endotoxin into the test sample. The detection range was from 0.010 to 0.100 EU mL^−1^, and then the IEC at a concentration of 0.05 EU mL^−1^ was used. The test was repeated twice for each sample and the test was repeated at least three times. A series of endotoxin standards with the concentration of 0.010–0.100 EU mL^−1^ are prepared, and the standard curve of endotoxin concentration and absorbance value is established.

### 2.21 Statistical Analysis

All data were expressed as the mean ± standard error of the mean. Statistical analyses were performed using one‐way analysis of variance (ANOVA) followed by Dunnett's multiple comparisons test. Calculations were performed using GraphPad Prism version 8.0 (Graphpad Prism Inc., San Diego, CA, USA). A P value of less than 0.05 was considered to indicate statistical significance.

## 3 Results

### 3.1 Preparation and Characterization of V_2_C QDs

**Figure 1:**
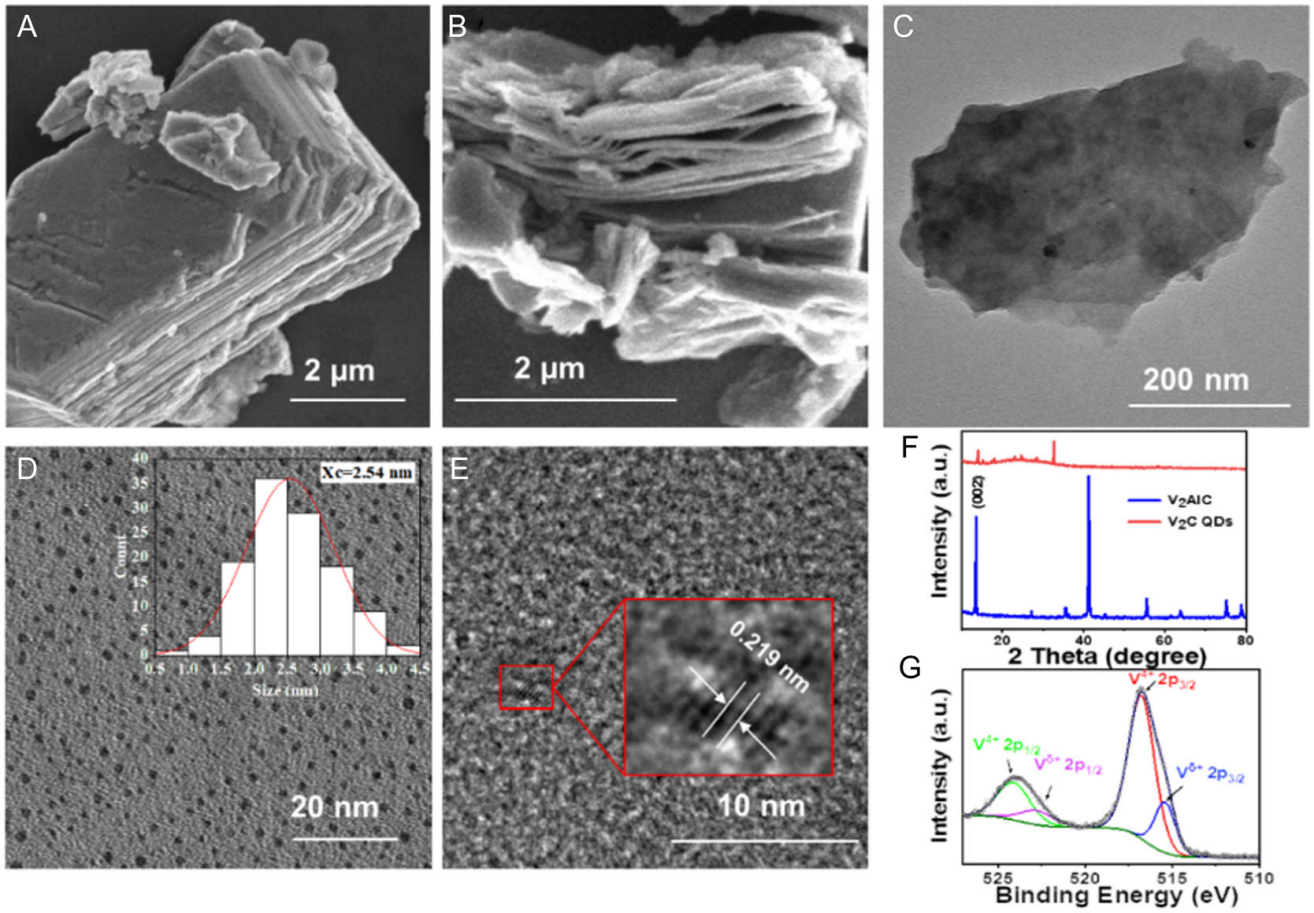
A) SEM images of V_2_AlC particles. B) Multilayer V_2_C and C) V_2_C nanosheets. D) TEM images, E) HRTEM images of V_2_C QDs, F) XRD spectra of V_2_C QDs, and G) XPS spectra of V_2_C QDs (0 < δ < 4).

V_2_C QDs was synthesized from V_2_AlC precursor by etching Al with HF (50%) aqueous solution according to our previous report with some modification.^[^ ^24^ ^]^ As shown in **Figure** **1**A,B, the structures and morphologies of V_2_AlC before and after HF etching were characterized using SEM, and the closed stratified structure of V_2_AlC changed to a typical accordion structure of V_2_C after Al etching. The element mapping clearly confirmed the elimination of Al layer during the HF etching process (Figure S1, Supporting Information). Then, the multilayer V_2_C was exfoliated in TPAOH under ultrasonic into 2D V_2_C nanosheets (Figure 1C). Furthermore, the 2D V_2_C QDs were obtained by a hydrothermal method (Figure 1D), which showed good dispersibility with an average size of ≈2.54 nm characterized by the TEM images (the size distribution was calculated by counting more than 100 nanoparticles). The hydrodynamic size of the QDs measured ≈7 nm (Figure S2, Supporting Information), consistent with TEM findings. As shown in Figure 1E, a set of lattice fringes with an inner plane spacing of 0.219 nm corresponded to the (121) plane of V_2_C was clearly observed in the zoomed‐in high‐resolution TEM (HRTEM) image. The typical X‐ray diffraction (XRD) pattern of V_2_AlC in agreement with PDF#29‐0101, and the V_2_C QDs showed main diffraction peaks located around 13.32°, suggesting the successful fabrication of V_2_C QDs.^[^ ^25^, ^29^ ^]^ The high‐resolution XPS spectra further indicated the successful fabrication of the V_2_C QDs (Figure 1G).^[^ ^24^ ^]^ These results confirmed the successful synthesis of V_2_C QDs.

### 3.2 The BBB Crossing Ability of V_2_C QDs

**Figure 2:**
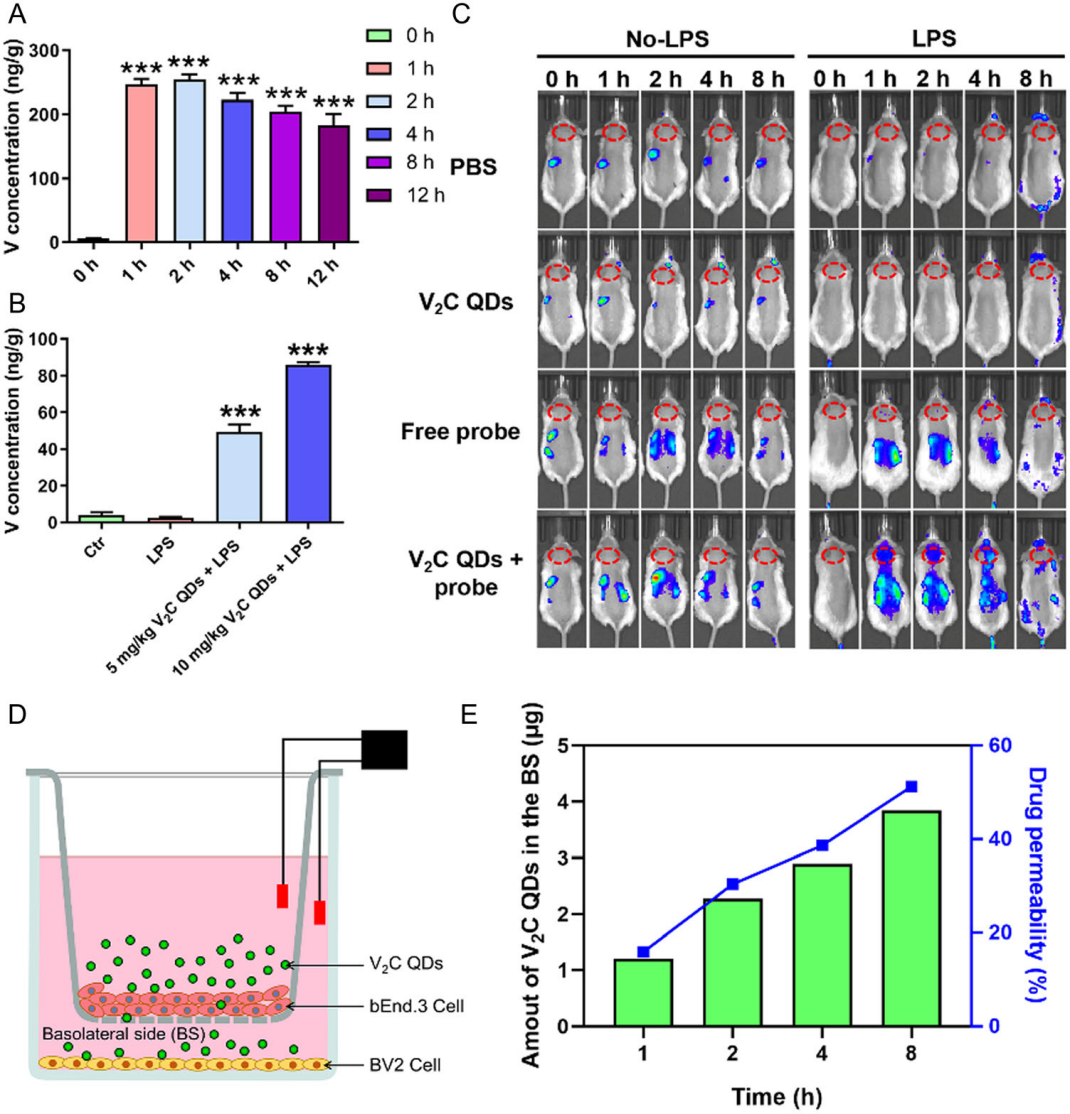
V_2_C QDs cross the blood–brain barrier. A) Vanadium levels in the brain of mice treated with 10 mg kg^−1^ V_2_C QDs were detected at different time points; *n* = 6 mice per group. ^***^ *P* < 0.001 for V_2_C QDs group versus untreated group. B) Vanadium levels in the brain of mice treated with V_2_C QDs (5 or 10 mg kg^−1^) or LPS were measured at 24 h post‐injection. C) Fluorescence images of mice with or without LPS stimulation collected at 0, 1, 2, 4, and 8 h posttreatment with PBS, V_2_C QDs, free miRNA‐34a probe or V_2_C QDs@probe (V_2_C dose, 10 mg kg^−1^, miRNA‐34a probe dose, 20 nmol kg^−1^); *n* = 6 mice per group. D) Schematic diagram of the in vitro BBB model. E) Transcytosis efficiency of V_2_C QDs measured by the in vitro BBB model. ***P < 0.001, LPS + V_2_C QD group versus LPS group.

Highly efficient BBB penetration and crossing ability are the prerequisite for the treatment of CNS disorders. Before investigating the BBB permeability of V_2_C QDs, the biocompatibility and physiological stability were first characterized. To determine the cytotoxicity of V_2_C QDs, we investigated the effect of V_2_C QDs on BV2 cells and bEnd.3 cells viability by conducting CCK‐8 assays. V_2_C QDs at different concentrations were applied to cultured BV2 cells and bEnd.3 cells. The assay results showed that V_2_C QDs concentrations ranging from 0 to 15 μg mL^−1^ had no cytotoxic effect on BV2 and bEnd.3 cells 24 h posttreatment (Figure S3, Supporting Information). Therefore, we selected four concentrations within this range (2, 5, 10, 15 μg mL^−1^) for using in subsequent experiments. The TEM images indicated the V_2_C QDs displayed good stability after being cultured in the cell medium for 12 and 24 h (Figure S4, Supporting Information). As inorganic nanomaterials, in addition to the biophysical effects that they exhibit, their biosafety requires special attention. Hence, we further evaluated the biosafety of V_2_C QDs. The hemolysis ratio is a vital parameter for assessing the biocompatibility of the biomaterials. A hemolysis ration less than 5% is acceptable, which meets the requirements of biomedical materials according to standard. As shown in Figure S5A,B, Supporting Information, obvious hemolysis had occurred in H_2_O group compared with PBS group. After treatment with V_2_C QDs, the hemolysis rate of these samples in the maximum dose group was still below 5%, indicating that these materials had good biocompatibility. Red blood cells are round and regular with no deformation and other unhealthy states under normal circumstances, while after hemolysis, it would shrink and rupture. Here, the microscopic morphology of red blood cells was also monitored. As shown in Figure S5C, Supporting Information, significant damage of red blood cells was observed in H_2_O group, while V_2_C QDs treatment did not affect the cell morphology. Then, we investigated the BBB permeability of V_2_C QDs using LPS‐stimulated mice. The brain uptake of vanadium was quantificationally determined by ICP–MS at different time points, and the results are shown in **Figure** **2**A,B. Vanadium levels in brain were gradually increased and reached most intense uptake at 2 h by treatment of V_2_C QDs, and then decreased owing to clearance from the body. Meanwhile, it displayed a concentration‐dependent uptake pattern (Figure 2A) and the accumulation of vanadium in 5 mg kg^−1^ V_2_C QDs‐treated and 10 mg kg^−1^ V_2_C QDs‐treated adult male mice still maintained at average 50 and 86 ng g^−1^ in the brain at 24 h post‐injection, respectively (Figure 2B). In addition, we conducted transwell assay by coculturing mouse brain microvascular endothelial (bEnd.3) cells and BV2 cells inside the apparatus, until the TEER exceeded 200 Ω cm^2^, indicating that the complete BBB in vitro model had been formed. Then, the V_2_C QDs were added into the upper chambers and evaluated the permeability of V_2_C QDs. As shown in Figure 2D,E, V_2_C QDs effectively transports across the bEnd.3 cells after 1, 2, 4, and 8 h incubation. To investigate the mechanism of BBB penetration, we also examined the localization of V_2_C QDs in BV2 cells by TEM in this study. As shown in Figure S6, Supporting Information, V_2_C QDs were found in the vesicle of the cell, suggesting the BBB penetration ability of V_2_C QDs may be dependent on cellular endocytosis. Such BBB permeability and clearance behavior of V_2_C QDs would be very attractive for CNS disorders treatment.

To monitor inflammation, we designed a probe to detect the microRNA (miRNA) miRNA‐34a (Figure S7A,B, Supporting Information), which has been found to be expressed at high levels in inflammatory diseases.^[^ ^30^, ^31^, ^32^ ^]^ The probe detected miRNA‐34a showed high selectivity (Figure S7C, Supporting Information) and sensitivity (Figure S8, Supporting Information). The miRNA‐34a detection probe was then assembled with the V_2_C QDs (V_2_C@probe) to investigate the in vivo diagnostic applicability of inflammation monitoring. LPS‐stimulated mice were divided into four groups according to the treatments they received: PBS, V_2_C QDs, free probe, or V_2_C@probe. The V_2_C@probe‐treated group showed a sustained increase in fluorescence, with maximum signal observed at 2 h post‐injection in the abdomen and head, while no obvious fluorescence increase was observed in the PBS‐treated or V_2_C QDs‐treated groups (Figure 2C). The negligible weak fluorescence observed in the free probe‐treated group suggested free probe barely crossed BBB. The fluorescence detected in the brains of V_2_C@probe‐treated mice confirmed the ability of the V_2_C QDs, to cross the BBB, as well as the capacity of the miRNA‐34a probe to diagnose inflammation.

To investigate the biodegradability of V_2_C QDs in vivo, the distribution of V_2_C QDs in each organ of mice was examined by ICP–MS, and the results showed that V_2_C QDs preferentially accumulated in the blood, brain, liver, lung, and kidney tissue 1 h post‐injection, while the content of vanadium in these tissues decreased markedly in 72 h, indicating that V_2_C QDs could effectively be scavenged in the body (Figure S9, Supporting Information). Therefore, it is reasonable to deduce that the V_2_C QDs in mice undergo a degradation process. In addition, we detected the endotoxin in V_2_C QDs by the LAL assay. As shown in Figure S10, Supporting Information, the endotoxin concentration was much lower than the standard set by the FDA, suggesting no endotoxin contamination at dose of 5 and 10 mg kg^−1^ V_2_C QDs was observed.

### 3.3 V_2_C QDs Ameliorate Spatial Learning and Memory Impairment in LPS‐Stimulated Mice

**Figure 3:**
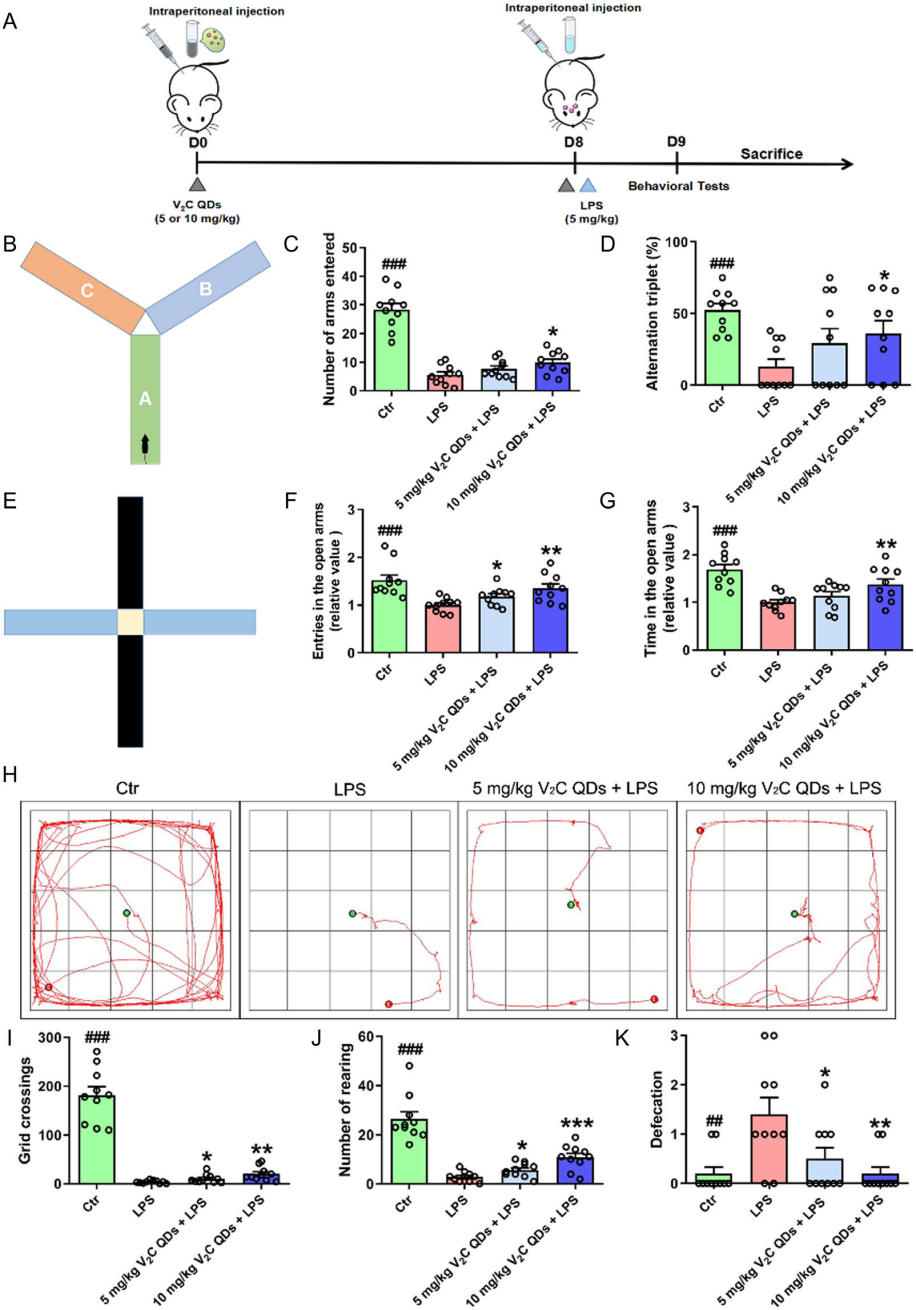
V_2_C QDs administration improves spatial learning and memory impairment in LPS‐stimulated mice. A) Scheme of V_2_C QDs treatment and behavioral tests. B) Illustration of the Y‐maze test. C, D) Numbers of arms entered and alternation triplets in the Y‐maze test. E) Schematic representation of the elevated plus maze test. F,G) Elevated plus maze test results, including entries into open arms and time spent in open arms during the experiment. H) Representative moving trails for mice in the open field during the 5 min test period. I–K) Numbers of crossed grids, time rearing, and frequencies of defecation in the open field test; *n* = 10 mice. ^*##*^ *P* < 0.01 and ^*###*^ *P* < 0.001, for control group (Ctr) versus LPS group; ^ *^ *P* < 0.05, ^**^ *P* < 0.01, and ^***^ *P* < 0.001 for LPS + V_2_C QDs group versus LPS group.

Using LPS‐stimulated mice as model, we investigated the anti‐inflammatory effects of V_2_C QDs. Following the administration of V_2_C QDs and LPS, three different behavioral tests were carried out to investigate depression, anxiety, learning, memory, and exploring ability in the treated mice (**Figure** **3**A). The Y‐maze test was performed to investigate the effect of the V_2_C QDs on learning and memory. During the test, the mice in the LPS group showed markedly fewer entries into the arms and fewer spontaneous alternations than the mice in the control group, indicating memory impairments in the LPS‐induced mice. Notably, following V_2_C QDs treatment, the LPS‐induced mice showed significant increases in both the numbers of entries into the arms and the percentages of spontaneous alternations (*P* < 0.05; Figure 3B–D). According to these outcomes, V_2_C QDs treatment significantly improved learning and memory deficits in LPS‐induced mice.

In addition, to investigate the antidepression and antianxiety effects of V_2_C QDs, the anxiety behaviors of mice in each group were assessed using the elevated plus maze test. The results showed that the numbers of entries and the time spent in the open arms were significantly decreased in LPS‐induced mice, compared with control mice. By contrast, V_2_C QDs treatment significantly increased these parameters in the open arms (^###^ *P* < 0.001; Figure 3E–G). To further evaluate the effect of V_2_C QDs on the exploration ability and anxiety behavior of LPS‐induced mice, we conducted the open field test. Compared with control mice, the numbers of grids crossed and rearing events were markedly decreased in the LPS‐induced mice (0.02 folds and 0.11 folds, ^*###*^ *P* < 0.001; Figure 3I,J), while defecation frequency was significantly increased (7 folds, ^*##*^ *P* < 0.01; Figure 3K). These findings confirmed typical depression‐ and anxiety‐like symptoms in the LPS‐induced mice. However, V_2_C QDs treatment significantly ameliorated these emotional disorders, enhancing the numbers of grid crossings (5.6 folds, ^**^ *P* < 0.01) and amount of rearing (3.7 folds, ^***^ *P* < 0.001) and reducing the frequency of defecation (0.14 folds, ^**^ *P* < 0.01; Figure 3H–K). Taken together, the results of these behavioral tests suggest that V_2_C QDs treatment can reduce the degree of anxiety and cognitive impairment in LPS‐induced mice.

### 3.4 V_2_C QDs Inhibit Glial Cell Overactivation in LPS‐Stimulated Mice

**Figure 4:**
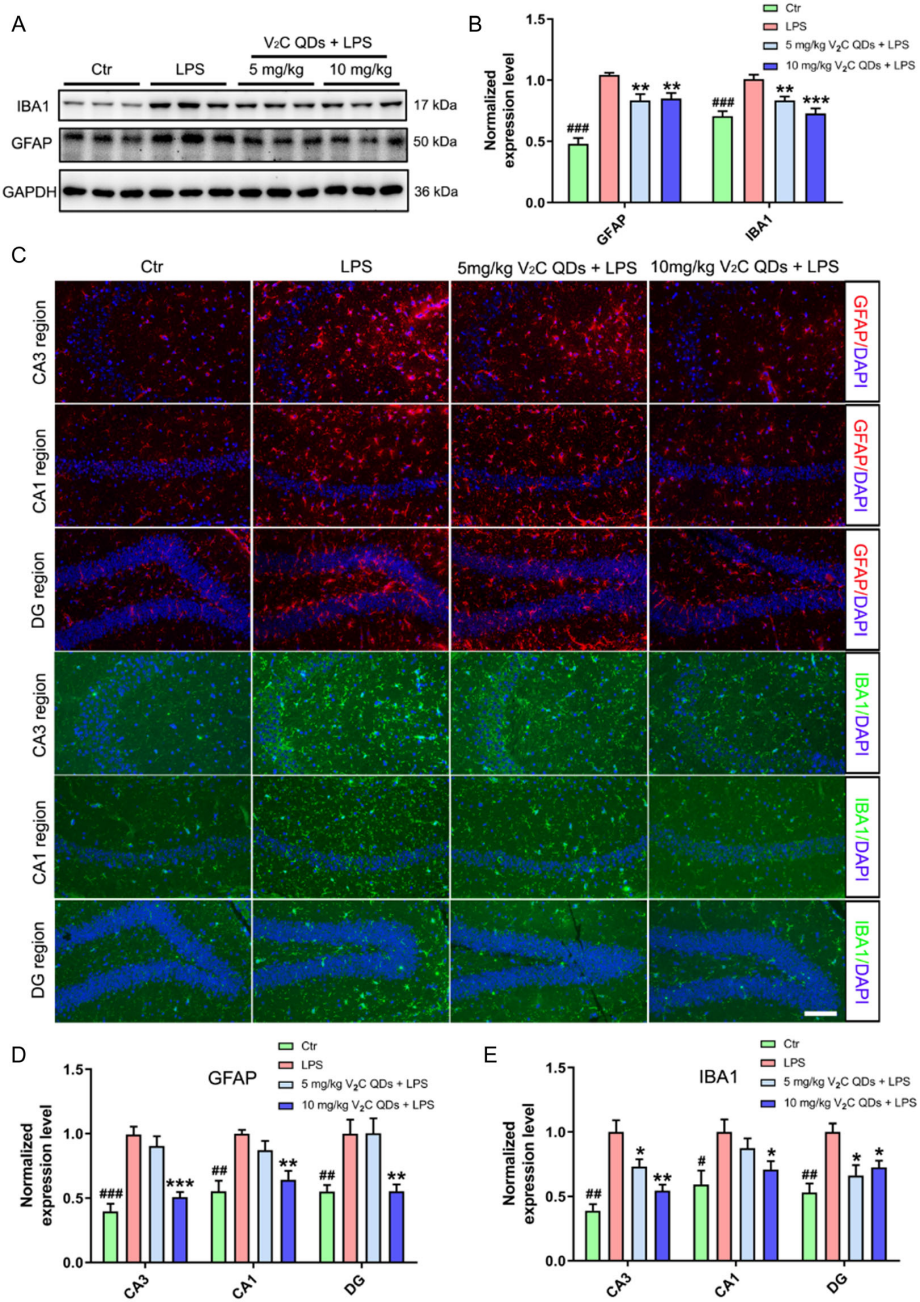
V_2_C QDs suppress glial activation in the hippocampus of LPS‐stimulated mice. A) Representative Western blots showing IBA1 and GFAP levels in the hippocampus. B) Quantitation of IBA1 and GFAP levels, normalized by glyceraldehyde‐3‐phosphate dehydrogenase (GAPDH). C) Representative immunofluorescent images showing activation of astrocytes and microglial cells, as indicated by staining of GFAP and IBA1 in the CA3, CA1, and DG regions of the hippocampus. D,E) The fluorescence intensity of GFAP (D) and IBA1 (E) was quantified using Image‐Pro Plus 6.0; *n* = 6 mice. ^*#*^ *P* < 0.05, ^*##*^ *P* < 0.01, and ^*###*^ *P* < 0.001, Ctr group versus LPS group; ^ *^ *P* < 0.05, ^**^ *P* < 0.01, and ^***^ *P* < 0.001, LPS + V_2_C QDs group versus LPS group. Scale bar: 100 μm.

Activation of microglia and astrocytes is implicated in the pathogenesis of cognitive decline in neuroinflammatory and NDs.^[^ ^1^, ^33^, ^34^, ^35^ ^]^ To evaluate the effects of V_2_C QDs on the activation of glial cells, we measured the levels of glial fibrillary acidic protein (GFAP) and ionized calcium‐binding adaptor molecule‐1 (IBA1) protein in the treated mice (**Figure** **4**A,B). These molecules are astrocyte‐ and microglial‐specific markers that are upregulated after microglia and astroglia activation.^[^ ^36^ ^]^ Western blot analysis of proteins extracted from mouse hippocampus showed significantly higher levels of GFAP and IBA1 in LPS‐stimulated mice than in control mice. By contrast, IBA‐1 and GFAP levels were significantly lower in mice pretreated with V_2_C QDs. Furthermore, immunofluorescence staining to detect GFAP and IBA1 in the brain showed strong GFAP and IBA1 signals in activated astrocytes and microglia within the hippocampus at 24 h after LPS injection (Figure 4C–E). V_2_C QDs treatment significantly repressed the increases in GFAP and IBA1 immunoreactivity in the cornu ammonis 3 (CA3), CA 1 (CA1), and dentate gyrus (DG) subregions of the hippocampus. These findings indicate that V_2_C QDs can inhibit the excessive activation of microglia and astrocytes.

### 3.5 V_2_C QDs Prevent the Production of Pro‐Inflammatory Mediators

**Figure 5:**
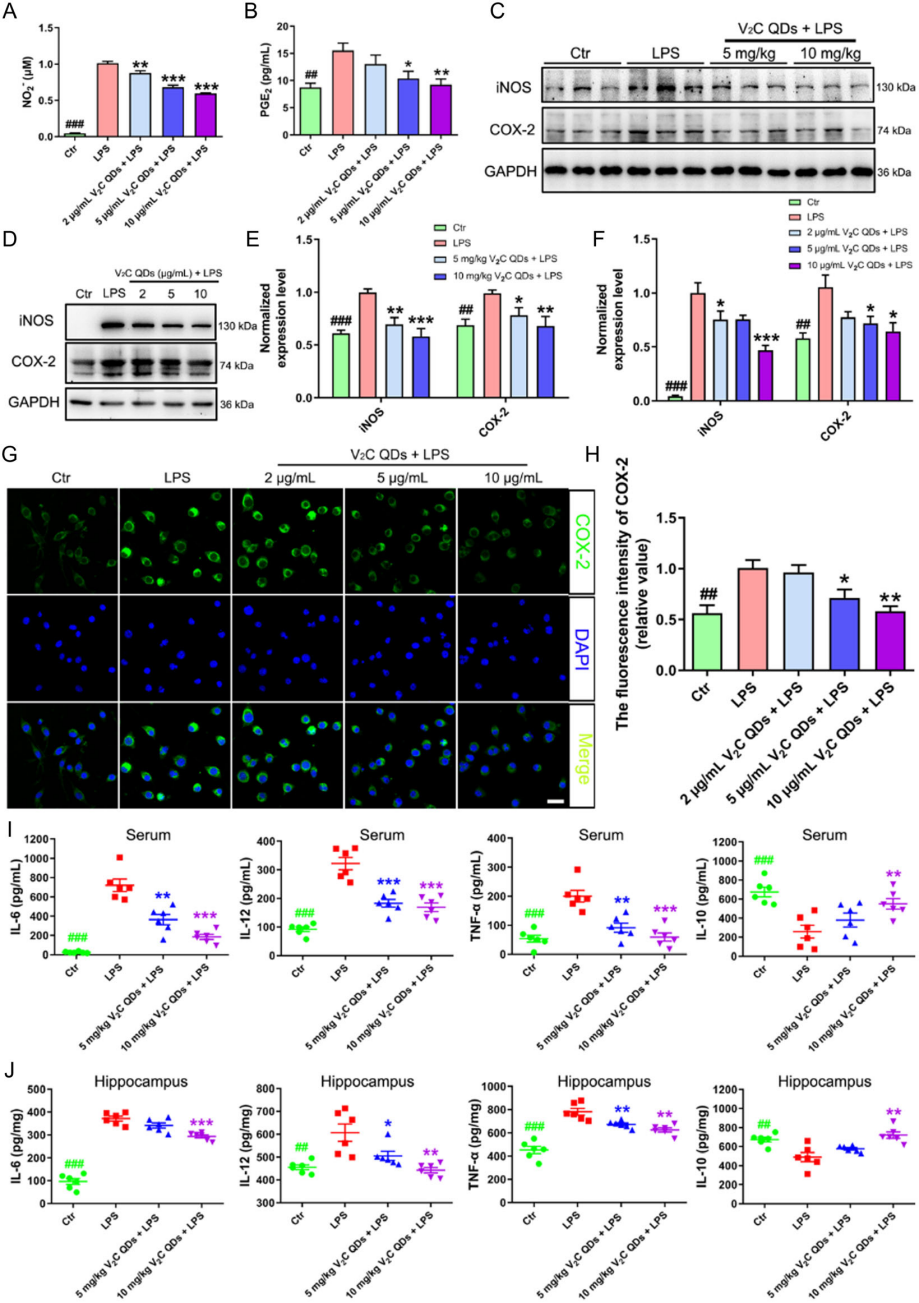
V_2_C QDs prevent the production of pro‐inflammatory mediators in LPS‐induced mice and BV2 cells. A,B) NO and PGE_2_ released to the supernatant of BV2 cell culture were measured using Griess reagent and ELISA assay, respectively. C,D) Representative western blots showing iNOS and COX‐2 levels in C) mouse hippocampus and D) BV2 cells. E,F) Quantitation of iNOS and COX‐2 protein levels in E) mouse hippocampus and F) BV2 cells, normalized by GAPDH. G) Representative immunofluorescent images showing the expression level of COX‐2 in BV2 cells. H) Quantitation of fluorescence intensity using Image‐Pro Plus 6.0; *n* = 3 experiments. I,J) The production of IL‐6, IL‐12, TNF‐α, and IL‐10 in I) the serum and J) hippocampus of mice was determined using ELISA; *n* = 6 mice. ^*##*^ *P* < 0.01 and ^*###*^ *P* < 0.001, Ctr group versus LPS group; ^ *^ *P* < 0.05, ^**^ *P* < 0.01, and ^***^ *P* < 0.001, LPS + V_2_C QDs group versus LPS group. Scale bar: 20 μm.

Having shown the suppressive effect of V_2_C QDs on inflammation‐induced activation of glial cells in vivo, we wanted to explore the effects of these nanomaterials further in living cells. For this purpose, we used LPS to stimulate the mouse microglia cell line BV2. To assess the anti‐inflammatory effects of V_2_C QDs, we investigated NO and PGE_2_ generation in LPS‐induced BV2 cells. NO and PGE_2_ are two vital inflammatory mediators that are released by over‐activated microglia during the neuroinflammatory response.^[^ ^37^ ^]^ After stimulation with LPS, the levels of NO and PGE_2_ in the cell culture supernatant were increased (^*##*^ *P* < 0.01 and ^*###*^ *P* < 0.001, respectively). Interestingly, prophylactic V_2_C QDs treatment significantly inhibited the excessive secretion of NO and PGE_2_ in the LPS‐induced cells (^**^ *P* < 0.01 and ^***^ *P* < 0.001, respectively; **Figure** **5**A,B). To further investigate the potential of V_2_C QDs in a therapeutic treatment setting and whether V_2_C QDs could reverse the damage already done by LPS, the BV2 cells were treated with V_2_C QDs after LPS exposure at different time points (0, 2, and 4 h). As shown in Figure S11, Supporting Information, V_2_C QDs reduced the levels of NO after LPS exposure, suggesting that V_2_C QDs could also reverse the damage already done by LPS and show effective therapeutic treatment.

The formation of NO and PGE_2_ is catalyzed by iNOS and COX‐2, respectively, in glial cells. Therefore, we went on to examine whether the expression levels of iNOS and COX‐2 could be regulated by V_2_C QDs in LPS‐induced mice and BV2 cells. By western blot analysis, the levels of iNOS and COX‐2 proteins were increased 1.7 folds and 1.4 folds in LPS‐induced mice (^##^ *P* < 0.01 and ^###^ *P* < 0.001, respectively) and 23 folds and 1.5 folds in BV2 cells (^##^ *P* < 0.01 and ^###^ *P* < 0.001, respectively), while V_2_C QDs treatment significantly decreased the levels of these proteins (Figure 5C–F). In addition, immunofluorescence staining showed that the fluorescence intensity of COX‐2 was higher in LPS‐stimulated BV2 cells than in untreated BV2 cells (^*##*^ *P* < 0.01) and was significantly reduced after V_2_C QDs treatment (^**^ *P* < 0.01; Figure 5G,H).

Next, to determine whether V_2_C QDs could affect the release of inflammatory cytokines in LPS‐stimulated mice, the levels of IL‐6, IL‐12, TNF‐α, and IL‐10 were measured using ELISA. The cytokines IL‐6, IL‐12, and TNF‐α are crucial pro‐inflammatory factors, while IL‐10 is an important anti‐inflammatory factor secreted by microglia.^[^ ^38^, ^39^ ^]^ Comparison with control mice showed that the levels of IL‐6, IL‐12, and TNF‐α were greatly increased and the level of IL‐10 was greatly reduced in both the brain (3.9 folds, 1.3 folds, 1.7 folds, and 0.7 folds) and serum (29 folds, 3.5 folds, 3.7 folds, and 0.4 folds) of LPS‐stimulated mice (^*##*^ *P* < 0.01 or ^*###*^ *P* < 0.001; Figure 5I,J). Treating LPS‐stimulated mice with V_2_C QDs significantly reduced the levels of IL‐6, IL‐12, and TNF‐α and enhanced the level of IL‐10. These in vivo observations were verified by similar results obtained in BV2 cells (Figure S12A–D, Supporting Information). Collectively, these findings indicated that V_2_C QDs can indeed ameliorate neuroinflammation by suppressing glial cell activation, reducing the release of pro‐inflammatory cytokines, and promoting the secretion of anti‐inflammatory cytokines.

### 3.6 V_2_C QDs Prevent the Upregulation of TLR4 and MyD88

**Figure 6:**
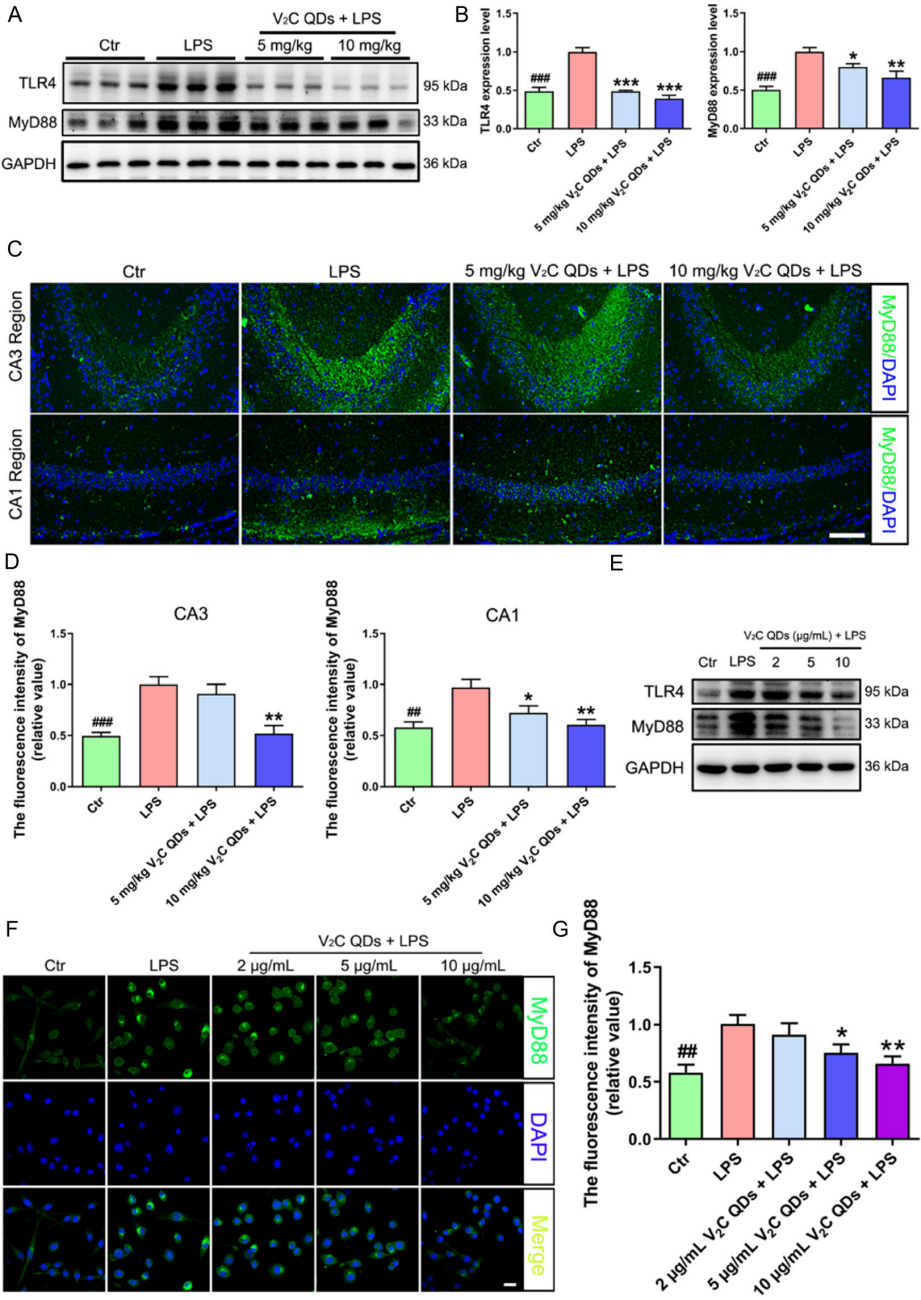
V_2_C QDs inhibit the upregulation of TLR4 and MyD88 in LPS‐induced mice and BV2 cells. A) Representative western blot analysis of TLR4 and MyD88 in mouse hippocampus. B) Quantitation of protein levels, normalized by GAPDH. C) Representative immunofluorescence images showing the level of MyD88 in mouse hippocampal CA3 and CA1 regions. D) Quantitation of MyD88 fluorescence intensity using Image‐Pro Plus 6.0; *n* = 6 mice. E) Representative western blot analysis of TLR4 and MyD88 in BV2 cells. F) Representative images showing MyD88 expression level in BV2 cells. G) Fluorescence intensity for MyD88 was quantified using Image‐Pro Plus 6.0; *n* = 3 experiments. ^*##*^ *P* < 0.01 and ^*###*^ *P* < 0.001, Ctr group versus LPS group; ^ *^ *P* < 0.05, ^**^ *P* < 0.01, and ^***^ *P* < 0.001, LPS + V_2_C QDs group versus LPS group. Scale bar: 20 or 100 μm.

The good anti‐inflammatory effects of V_2_C QDs motivated us to investigate the molecular mechanism. TLR4, which recognizes LPS, is the most studied TLR on cell membranes. Specifically, following stimulation of TLR4 by LPS, MyD88 immediately binds to the cytoplasmic domain of TLR4, inducing the activation of the pro‐inflammatory signaling cascade.^[^ ^8^, ^40^ ^]^ To clarify the anti‐inflammatory mechanism of V_2_C QDs, we examined the levels of TLR4 and MyD88 in the hippocampus of LPS‐stimulated mice and LPS‐stimulated BV2 cells via western blot analysis (**Figure** **6** and S13, Supporting Information). The results showed that the levels of TLR4 and MyD88 proteins were significantly higher in the LPS groups than in the control groups. V_2_C QDs intervention resulted in significantly decreased TLR4 and MyD88 levels both in vivo (0.8 folds and 0.4 folds; ^***^ *P* < 0.001 and ^**^ *P* < 0.01, respectively) and in vitro (0.4 folds and 0.7 folds; ^ *^ *P* < 0.05 and ^***^ *P* < 0.001, respectively). In addition, immunofluorescence staining was performed to verify the effects of V_2_C QDs treatment on MyD88 expression. Consistent with the western blot results, the immunoreactivity of MyD88 was markedly higher in the CA3 and CA1 hippocampal regions of LPS‐stimulated mice and in LPS‐stimulated BV2 cells than in control mice or BV2 cells, while V_2_C QDs treatment significantly reduced MyD88 immunoreactivity (Figure 6C,D,F,G). These findings indicate that inhibition of TLR4/MyD88 signaling may be prominently associated with the anti‐inflammatory properties of V_2_C QDs.

### 3.7 V_2_C QDs Suppress the Activation of NF‐κB‐ and MAPK‐Signaling Pathways

**Figure 7:**
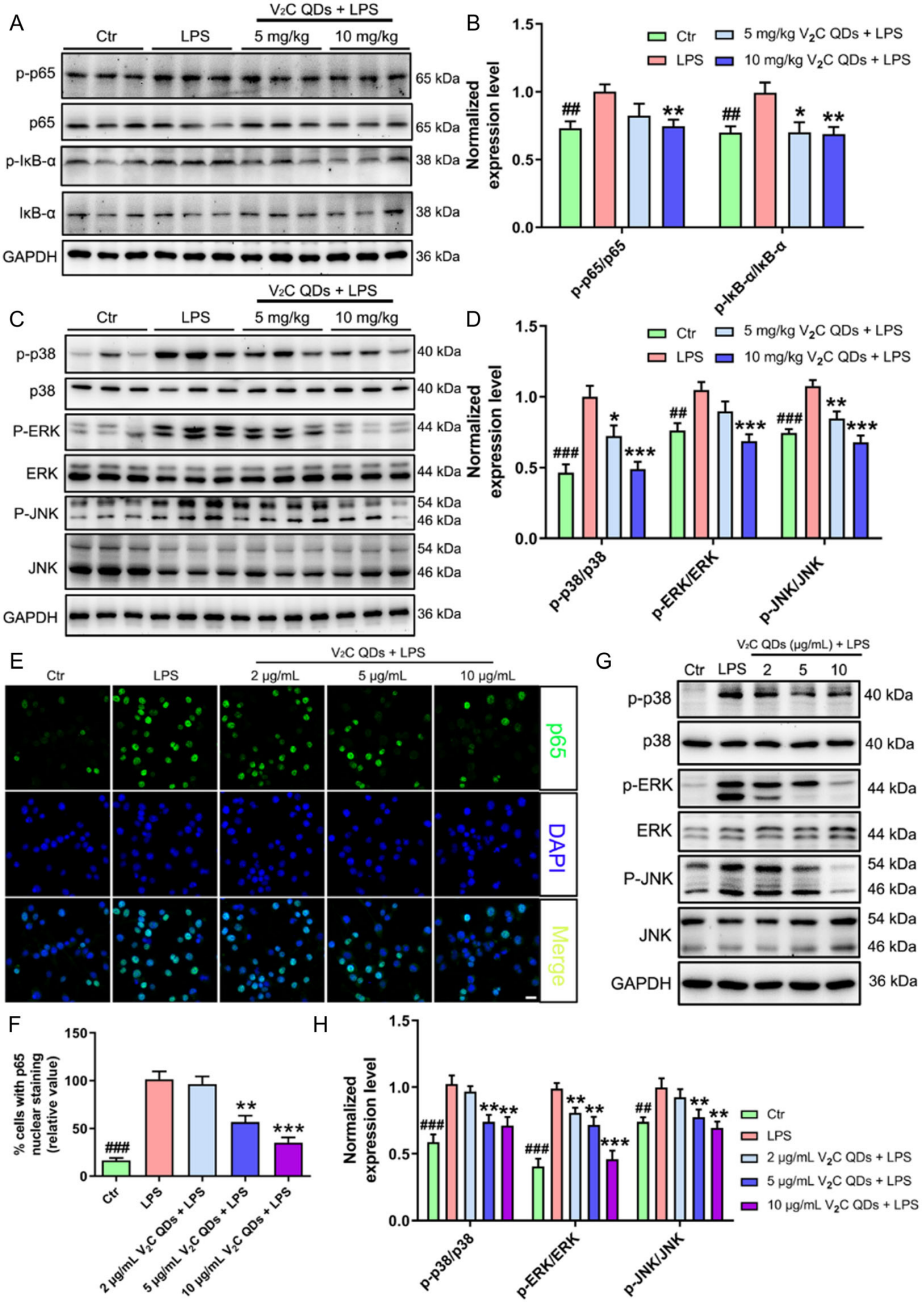
V_2_C QDs suppress LPS‐induced activation of the NF‐κB‐ and MAPK‐signaling pathways. A) Representative western blot analysis of p‐p65, p65, p‐IκB‐α, and IκB‐α in mouse hippocampus; B) the corresponding normalized p‐p65/p65 and p‐IκB‐α/IκB‐α ratios. C) Representative western blot analysis of p‐p38, p38, p‐ERK, ERK, p‐JNK, and JNK in mouse hippocampus; D) the corresponding normalized p‐p38/p38, p‐ERK/ERK, and p‐JNK/JNK ratios; *n* = 6 mice. E) Representative immunofluorescent images showing nuclear translocation of the NF‐κB/p65 subunit in BV2 cells. F) Nuclear translocation of the NF‐κB/p65 subunit was quantitated using Image‐Pro Plus Version 6.0. G) Representative western blot analysis of p‐p38, p38, p‐ERK, ERK, p‐JNK, and JNK in BV2 cells and H) the corresponding normalized protein ratios; *n* = 6 experiments. ^*##*^ *P* < 0.01 and ^*###*^ *P* < 0.001, Ctr group versus LPS group; ^ *^ *P* < 0.05, ^**^ *P* < 0.01, and ^***^ *P* < 0.001, LPS + V_2_C QDs group versus LPS group. Scale bar: 20 μm.

In various cell types, including microglia, LPS‐induced activation of TLR4 results in MyD88‐dependent phosphorylation of the NF‐κB/p65 subunit. This subunit is then translocated to the nucleus, where it regulates the generation of inflammatory cytokines. To explore the potential link between the anti‐inflammatory effect of V_2_C QDs and the TLR4/MyD88/NF‐κB‐signaling pathway, the levels of p‐p65, p65, p‐IκB‐α, and IκB‐α were examined in LPS‐induced mice and BV2 cells by western blot analysis. As expected, the levels of phosphorylated p65 and IκB‐α were higher in the LPS groups than in the control groups. Moreover, V_2_C QDs treatment restored the ratios of p‐p65/p65 and p‐IκB‐α/IκB‐α to control levels in both LPS‐induced mouse hippocampus (^##^ *P* < 0.01; **Figure** **7**A,B) and LPS‐stimulated BV2 cells (^###^ *P* < 0.001 and ^##^ *P* < 0.01; Figure S14A–C, Supporting Information). We also performed immunofluorescent staining to examine the effect of V_2_C QDs on the nuclear translocation of the NF‐κB p65 subunit. In unstimulated BV2 cells, p65 was primarily localized in the cytoplasm (Figure 7E,F), but following LPS stimulation, it was translocated into the nucleus. V_2_C QDs treatment significantly inhibited NF‐κB/p65 nuclear translocation in LPS‐induced BV2 cells.

As well as activating NF‐κB signaling, LPS activates another important pathway downstream of TLR4/MyD88, the MAPK pathway. MAPK family members include p38, extracellular signal‐regulated kinases (ERKs), and c‐Jun amino‐terminal kinases (JNKs). To investigate the potential involvement of the MAPK‐signaling pathway in the anti‐inflammatory effects of V_2_C QDs, the levels of p‐p38, p38, p‐ERK, ERK, p‐JNK, and JNK proteins were measured in LPS‐induced mice and BV2 cells by western blotting analysis. The results showed that the levels of phosphorylated p38, ERK, and JNK were significantly increased following LPS treatment, while V_2_C QDs treatment reduced the levels of p‐p38, p‐ERK, and p‐JNK back to near control levels in both LPS‐induced mice (^***^ *P* < 0.001; Figure 7C,D) and LPS‐stimulated BV2 cells (**P < 0.01 or ^***^ *P* < 0.001; Figure 7G,H). Taken together, these results indicate that suppression of LPS‐induced activation of TLR4/MyD88/NF‐κB‐ and TLR4/MyD88/MAPK‐signaling pathways may be associated with the inhibitory effect of V_2_C QDs on inflammatory molecules.

### 3.8 V_2_C QDs Suppress ER Stress in LPS‐Stimulated Mice and BV2 Cells

**Figure 8:**
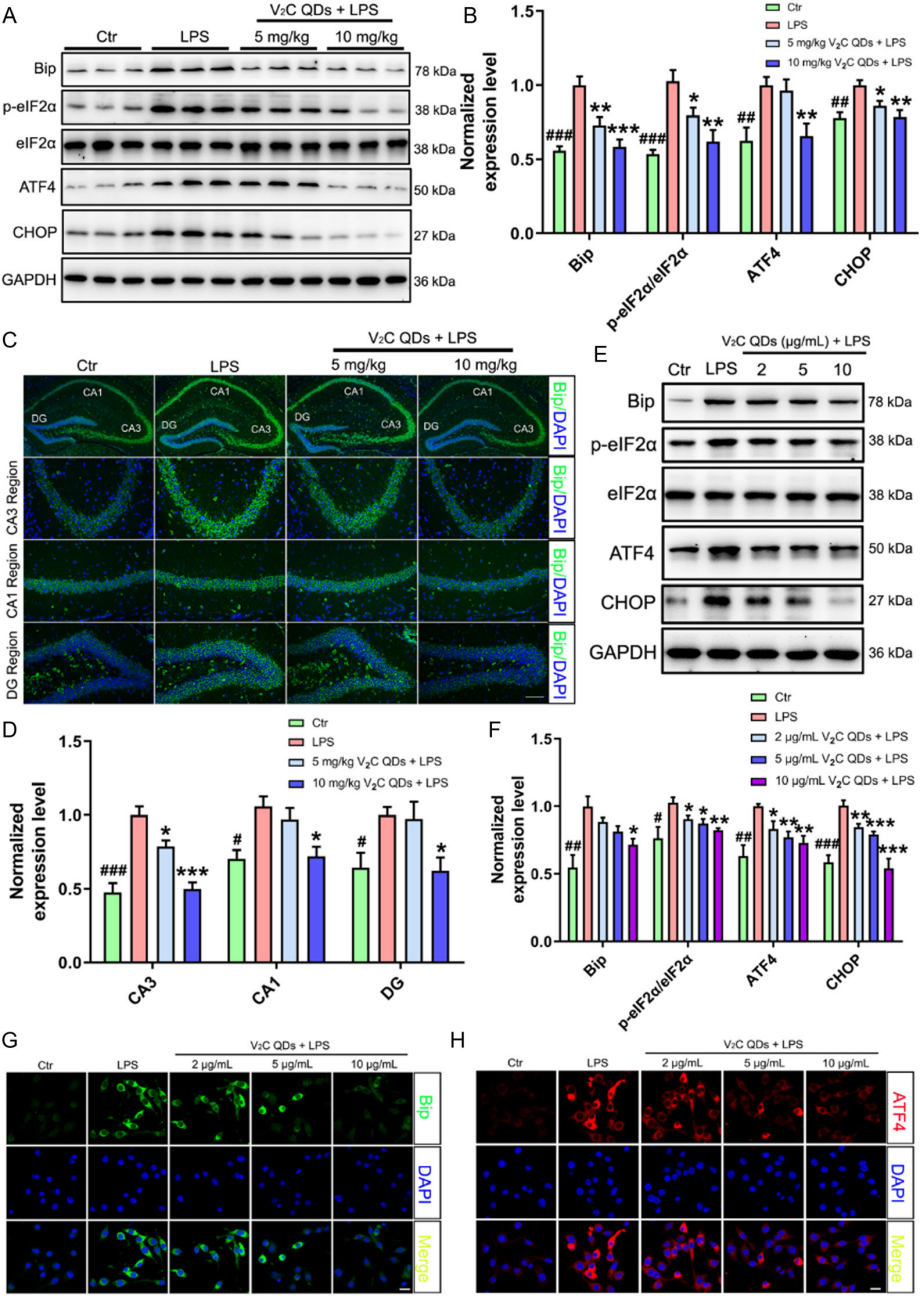
V_2_C QDs suppress ER stress in LPS‐stimulated mice and BV2 cells. A) Representative western blot analysis of Bip, p‐eIF2α, eIF2α, ATF4, and CHOP in mouse hippocampus. B) Quantitation of normalized proteins. C) Representative immunofluorescent images showing the levels of Bip protein in the CA3, CA1, and DG regions of the hippocampus. D) Quantitation of Bip fluorescence intensity using Image‐Pro Plus 6.0; *n* = 6 mice. E) Representative western blot analysis of Bip, p‐eIF2α, eIF2α, ATF4, and CHOP in BV2 cells. F) Quantitation of normalized protein levels. G,H) Representative immunofluorescent images showing the expression level of G) Bip and H) ATF4 in BV2 cells (*n* = 3 experiments). ^*#*^ *P* < 0.05, ^*##*^ *P* < 0.01, and ^*###*^ *P* < 0.001, Ctr group versus LPS group; ^ *^ *P* < 0.05, ^**^ *P* < 0.01, and ^***^ *P* < 0.001, LPS + V_2_C QDs group versus LPS group. Scale bar: 20 or 100 μm.

Endocytosis is an important way for nanomaterials to enter cells. To investigate whether V_2_C QDs can be endocytosed by BV‐2 cells, we examined the localization of V_2_C QDs in BV2 cells by TEM in this study. As shown in Figure S6 (Supporting Information), V_2_C QDs were found in the vesicle of the cell and the vesicles coated with V_2_C QDs fused with the ER, suggesting that V_2_C QDs located in the ER and exerted its biological functions. As LPS has been reported to induce ER stress, which plays a vital role in regulating inflammatory responses,^[^ ^41^ ^]^ we wanted to determine whether V_2_C QDs might exert an inhibitory effect on LPS‐induced ER stress in mouse hippocampus. Thus, we examined the levels of the classical ER stress‐associated proteins Bip, ATF4, and CHOP and the activation status of p‐eIF2α in LPS‐ and V_2_C QDs‐treated mice by western blot. The results showed that the levels of Bip, ATF4, and CHOP proteins and phosphorylated p‐eIF2α were significantly increased in mouse hippocampus following LPS treatment (**Figure** **8**A,B). By contrast, pretreatment with V_2_C QDs led to significant reductions in the LPS‐induced Bip (^***^ *P* < 0.001), ATF4 (^**^ *P* < 0.01), and CHOP (^**^ *P* < 0.01) levels, as well as the p‐eIF2α/eIF2α ratio (^**^ *P* < 0.01). In agreement with these findings, immunofluorescence staining revealed markedly increased levels of Bip protein in the CA1, CA3, and DG hippocampal regions following induction with LPS, which were significantly reduced by V_2_C QDs treatment (^###^ *P* < 0.001 or ^#^ *P* < 0.05; Figure 8C,D). Similar western blotting results were observed in BV2 cells, as well as a dose‐dependent effect of V_2_C QDs on CHOP, p‐eIF2α, and eIF2α levels and the p‐IFf2α/eIf2α ratio (Figure 8E,F). Immunofluorescence analysis further confirmed that V_2_C QDs treatment markedly decreased the fluorescence intensity of Bip (^**^ *P* < 0.01; Figure 8G and S15A, Supporting Information) and ATF4 (^**^ *P* < 0.01; Figure 8H and S15B, Supporting Information) in BV2 cells. In summary, our results suggest that V_2_C QDs can inhibit LPS‐induced ER stress both in vivo and in vitro.

### 3.9 V_2_C QDs Inhibit Oxidative Stress in LPS‐Stimulated Mice and BV2 Cells

**Figure 9:**
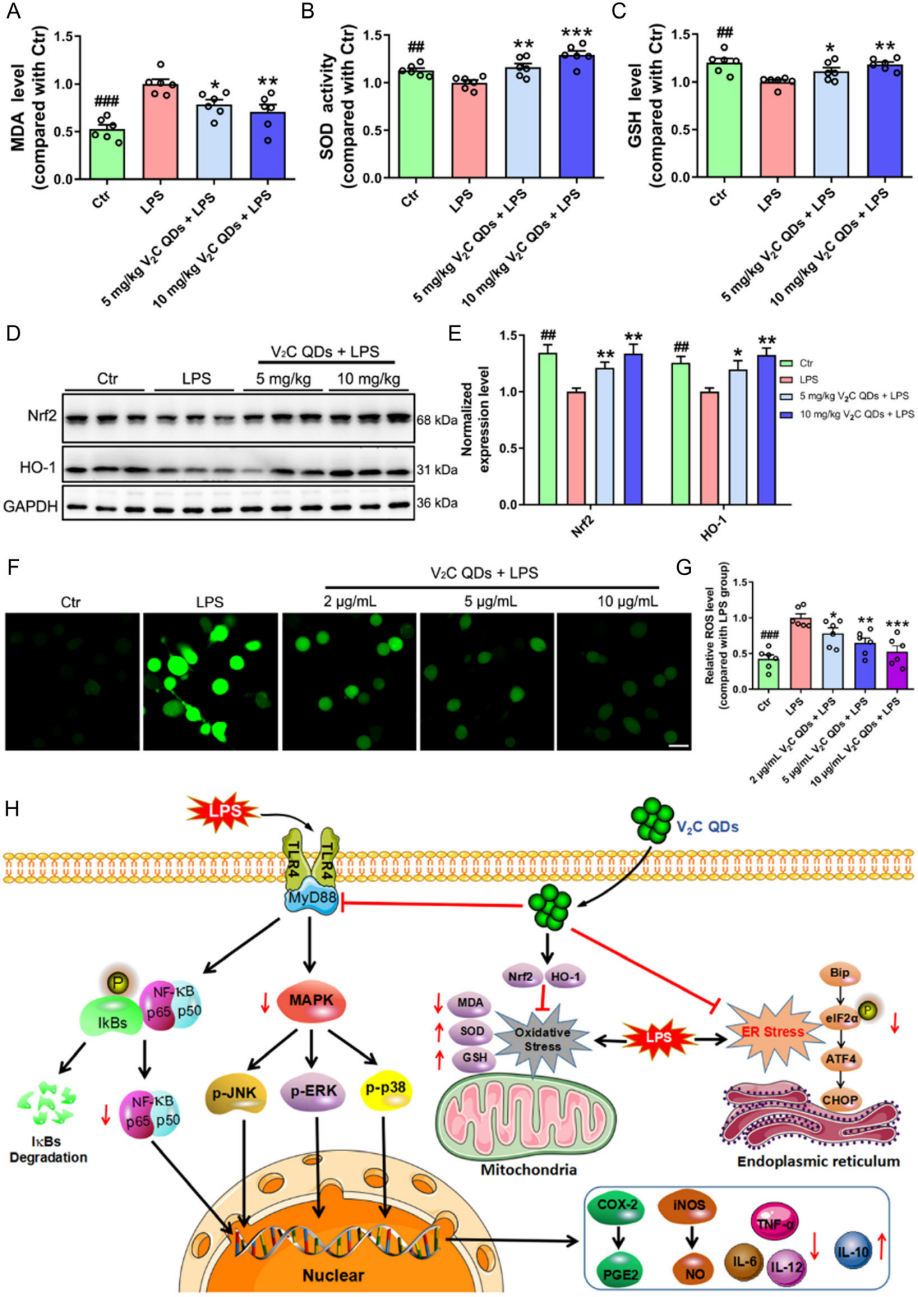
V_2_C QDs inhibit oxidative stress in LPS‐stimulated mice and BV2 cells. A–C) The relative concentrations of A) MDA and C) GSH and B) levels of SOD activity in the brains of mice in the Ctr, LPS, and V_2_C QDs groups were measured using assay kits. D) Representative Western blot analysis of Nrf2 and HO‐1 in mouse hippocampus. E) Quantitation of protein levels, normalized by GAPDH (*n* = 6 mice). F) Representative ROS staining in BV2 cells detected with DCFH–DA fluorescence probe. G) Quantitation of relative ROS levels. H) Proposed mechanisms of the protective effects of V_2_C QDs on LPS‐induced neuroinflammation, ER stress, and oxidative stress. *n* = 3 experiments. ^*##*^ *P* < 0.01 and ^*###*^ *P* < 0.001, Ctr group versus LPS group; ^ *^ *P* < 0.05, ^**^ *P* < 0.01, and ^***^ *P* < 0.001, LPS + V_2_C QDs group versus LPS group. Scale bar: 20 μm.

Many studies have shown that oxidative stress caused by stimulation with LPS plays a critical role in inflammatory processes.^[^ ^42^ ^]^ MDA, a major product during the process of lipid peroxidation, is regarded as an important indicator of oxidative stress,^[^ ^43^ ^]^ while SOD and GSH are the two main antioxidants for free radical scavenging in vivo.^[^ ^44^, ^45^ ^]^ To investigate whether V_2_C QDs could inhibit LPS‐induced oxidative stress, we detected SOD activity and determined MDA and GSH concentrations in the brain of LPS‐stimulated mice. Comparing LPS‐stimulated mice and control mice showed that LPS treatment significantly reduced SOD activity (^##^ *P* < 0.01) and GSH levels (^##^ *P* < 0.01) and significantly increased MDA levels (^###^ *P* < 0.001) in mouse hippocampus (**Figure** **9**A–C). V_2_C QDs treatment significantly increased both SOD activity and GSH levels but suppressed the level of MDA.

To further explore the mechanism underlying the antioxidant effects of V_2_C QDs, we determined the levels of Nrf2 and HO‐1 protein in LPS‐stimulated mice. The Nrf2/HO‐1 pathway has a critical role in the regulation of oxidative stress: HO‐1 is an anti‐oxidative enzyme, and Nrf2 is a transcriptional activator of antioxidant response elements in response to oxidative stress.^[^ ^46^, ^47^ ^]^ Western blotting showed that LPS treatment resulted in significant decreases in Nrf2 and HO‐1 levels, while V_2_C QDs treatment largely reversed these effects (^**^ *P* < 0.01; Figure 9D,E). Next, as excess ROS production leading to oxidative stress is also closely associated with the inflammatory response, we investigated ROS production in BV2 cells using fluorescence microscopy. Comparison of LPS‐induced BV2 cells and control cells showed that ROS levels were significantly increased (^*###*^ *P* < 0.001) in the cells treated with LPS. Consistent with our previous results, treatment with different doses of V_2_C QDs significantly suppressed the production of intracellular ROS (^***^ *P* < 0.001; Figure 9F,G). Meanwhile, similar to the in vivo results, V_2_C QDs also significantly reduced MDA levels and enhanced GSH expression and SOD activity in LPS‐stimulated BV2 cells (Figure S16A–C, Supporting Information). In addition, analysis of Nrf2 and HO‐1 expression in LPS‐stimulated BV2 cells revealed significantly reduced levels of protein, compared with the levels in control cells. As expected, V_2_C QDs treatment significantly increased the protein levels of Nrf2 and HO‐1 in vitro (^**^ *P* < 0.01; Figure S16D–F, Supporting Information). To conclude, our results indicated that V_2_C QDs can ameliorate LPS‐induced oxidative stress by suppressing ROS production and activating the Nrf2/HO‐1‐antioxidant‐signaling pathway both in vivo and in vitro.

## 4 Discussion

Activation of microglia, leading to the release of a variety of inflammatory mediators, is suggested to play a critical role in the pathogenesis of various neurodegenerative disorders.^[^ ^48^, ^49^, ^50^ ^]^ Accumulated evidence suggests that blocking microglial activation may be a useful therapeutic strategy to combat inflammation‐related diseases.^[^ ^51^ ^]^ Vanadium, a vital biological trace element, regulates multiple physiological activities in various organs.^[^ ^52^, ^53^, ^54^ ^]^ However, there are also concerns surrounding its off‐target effect and the potential toxicity for its long‐term use in humans, which hinder the progress of vanadium compounds in clinical trials. Despite current misgivings, interests in developing these chemicals continue and many believe that they could still have therapeutic potential for the untreatable disease such as AD. V_2_C QDs, a typical vanadium carbide nanostructure, could be coated with nucleus‐target peptides and packaged into Arg–Gly–Asp‐engineered exosomes (Ex‐RGD) to form a V_2_C–TAT@Ex‐RGD versatile theranostic platform. V_2_C–TAT@Ex‐RGD could realize multimodal imaging‐guided cell nucleus‐targeted photothermal therapy in the near‐infrared biowindow at low temperature and induce no significant side effects or toxicities, indicating good potential for further biomedical clinical applications.^[^ ^24^ ^]^ Moreover, as demonstrated in both inflammation and neurodegeneration animal models, 2D V_2_C MXenzyme rebuilds the redox homeostasis without perturbing the endogenous antioxidant status and relieves ROS‐induced damage with benign in vivo therapeutic effects and is expected to be developed into a clinical drug for multifarious inflammation and neurodegeneration treatment.^[^ ^22^ ^]^ Therefore, with new technologies for drug delivery constantly being available, the side effect of vanadium compounds could become avoidable, raising the opportunity for development of truly effective and safe drugs for clinical application. In the present study, we sought to further understand whether V_2_C QDs could combat LPS‐induced neuroinflammation both in vivo and in vitro and to clarify the mechanisms underlying these effects. We demonstrated multiple lines of evidence showing that V_2_C QDs reduced the generation of inflammatory cytokines by inhibiting TLR4/MyD88‐mediated NF‐κB and MAPKs pathways, prevented oxidative stress by activating the Nrf2/HO‐1 pathway, and suppressed the ER stress by inhibiting eIF2α/ATF4/CHOP pathway, resulting in repressing the activation of glial cell. Moreover, behavioral tests showed that V_2_C QDs ameliorated the spatial learning and memory impairment of LPS‐induced mice.

The ability of nanoparticles to cross the BBB is a checkpoint for developing drugs to treat brain diseases. In a previous study, we confirmed that bis(ethylmaltolato)oxidovanadium (IV) was able to cross the BBB in APPswe/PS1E9 transgenic mice. Similarly, in the current study, we found that V_2_C QDs could also pass efficiently through the BBB into the brain. Therefore, we further adopted a mouse model of LPS‐induced inflammation to investigate the effects of V_2_C QDs. Compelling evidence has shown that LPS treatment in mice is able to cause microglial activation and upregulate pro‐inflammatory molecules.^[^ ^8^ ^]^ Consistent with these reports, LPS significantly activated microglia in our model, and V_2_C QDs effectively reduced the expression of the astrocyte‐specific and microglial‐specific markers IBA1 and GFAP in the hippocampus of LPS‐induced mice. These findings support the speculation that V_2_C QDs can pass through the BBB to inhibit glial cell activation in the brain and improved the exploration ability and memory ability of LPS‐induced mice, which were consistent with the previous reports that vanadium compounds can enter the brain and improve the cognitive deficits of AD mice.

As for the molecular mechanism research, we showed that V_2_C QDs treatment counteracted the effects of LPS on these inflammatory mediators including TNF‐α, IL‐6, IL‐12, IL‐10, NO, and PGE_2_ and enzymes in vivo and in vitro. These results are consistent with previous reports that vanadium compounds can suppress the release of inflammatory mediators.^[^ ^53^ ^]^ Our findings revealed that both the TLR4/MyD88/MAPKs‐ and TLR4/MyD88/NF‐κB‐signaling pathways were greatly activated in LPS‐induced mice and BV2 cells, whereas V_2_C QDs remarkably downregulated these signaling pathways. These results indicate that V_2_C QDs may prevent proinflammatory factors production by suppressing signal transduction via the NF‐κB and MAPK pathways. Meanwhile, V_2_C QDs significantly inhibited eIF2α/ATF4/CHOP signaling. Links between ER stress, microglial activation, and neuroinflammation have been demonstrated.^[^ ^55^ ^]^ Thus, we speculate that V_2_C QDs might also reduce neuroinflammation via reducing ER stress. Furthermore, we showed that treatment with V_2_C QDs effectively counteracted the effects of LPS on these parameters‐related to oxidative stress, suggesting that V_2_C QDs have an inhibitory effect on LPS‐induced oxidative stress, demonstrating that the effects of LPS and V_2_C QDs on oxidative stress in vivo and in vitro may be mediated via opposing effects on the Nrf2/HO‐1‐signaling pathway.

## 5 Conclusion

In summary, we have successfully synthesized an ultrasmall 2D V_2_C QDs with versatile BBB permeability and multiple biological functions. The 2D V_2_C QDs not only was capable of efficiently and noninvasively crossing BBB, but also delivering gene probe to the brain for neuroinflammation diagnosis. Our data clearly indicated, for the first time, that V_2_C QDs remarkably attenuated neuroinflammation, ER stress, and oxidative stress induced by LPS, ultimately ameliorating learning and memory impairment in a mouse model system. Mechanism studies further elucidated that V_2_C QDs exerted their effects by inhibiting NF‐κB, MAPK, and eIF2α/ATF4/CHOP signaling, and activating Nrf2/HO‐1‐signaling pathways (Figure 9H). The mechanism by which QDs enter neuronal cells is not yet clear in current research. Hence, effective strategy may need to verify the specific internalization and degradation processes and mechanisms of V_2_C QDs in future study. Taken together, these results demonstrate that V_2_C QD is a promising candidate for treating neuroinflammation and its associated diseases. It also provides fascinating insights into the design and application of highly efficient nanomaterials for neuroprotection and cerebropathy treatment.

## Conflict of Interest

The authors declare no conflict of interest.

## Author Contributions

**Haifeng Dong**, **Qiong Liu**, and **Zhijun He** designed the research. **Qiqi Yang** prepared and characterized the nanomaterial. **Zi Wang**, **Shengwu Wen**, **Ming-Jie Dong**, **Youcong Gong**, and **Zijia Zhou** took part in material analysis. **Zhijun He** and **Xiaoqian Li** performed the animal and cell experiments and analyzed the data. **Haifeng Dong**, **Qiong Liu**, **Zhijun He**, and **Qiqi Yang** cowrote the manuscript. All authors reviewed and concurred with the final manuscript.

## Supporting information

Supplementary Material

## Data Availability

The data that support the findings of this study are available in the supplementary material of this article.
