# Correction to “Vanadium Carbide Quantum Dots Exert Efficient Anti‐Inflammatory Effects in Lipopolysaccharide‐Induced BV2 Microglia and Mice”

**DOI:** 10.1002/smsc.70372

**Published:** 2026-08-19

1

Zhijun He, Qiqi Yang, Xiaoqian Li, Zi Wang, Shengwu Wen, Ming‐Jie Dong, Weiyun Zhang, Youcong Gong, Zijia Zhou, Qiong Liu,* Haifeng Dong* Small Sci. 2024, 4(10), 2300334, https://doi.org/10.1002/smsc.202300334

In the published article, the authors regret that an inadvertent error was found in Figure 8A,B. The error occurred during the assembly of the figure panels, when incorrect blot images of ATF4 were inadvertently inserted. We are submitting the corrected figure containing the ATF4 Western blot image and its quantitation results in the updated Figure 8A,B.

**Figure 8:**
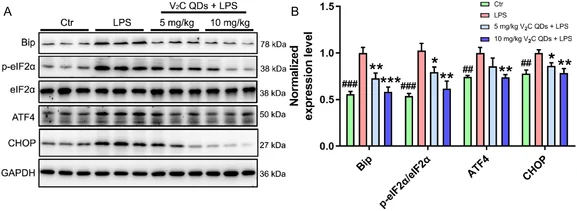
Updated Figure 8A and B.

We sincerely apologize for this oversight. These corrections do not alter any of the findings or conclusions of the paper.

