# Supplementary material for: Vanadium Carbide Quantum Dots Exert Efficient Anti‐Inflammatory Effects in Lipopolysaccharide‐Induced BV2 Microglia and Mice
Source: Small Sci. 2024 Sep 10;4(10):2300334. doi: 10.1002/smsc.202300334 (PMC11935053; doi:10.1002/smsc.202300334)
Supplement: Supplementary file 1 — Supplementary Material [file SMSC-4-2300334-s001.pdf]

Supplementary Materials

Vanadium Carbide Quantum Dots Exert Efficient Anti-Inflammatory Effects in Lipopolysaccharide-induced BV2 Microglia and Mice

Zhijun He[b,c#], Qiqi Yang[a,e#], Xiaoqian Li[b#], Zi Wang[b], Shengwu Wen[a], Ming-Jie Dong[f], Weiyun Zhang[a], Youcong Gong[a], Zijia Zhou[a], Qiong Liu[b,d]*, Haifeng Dong[a]*

[a] Marshall Laboratory of Biomedical Engineering, Precision Medicine and Health Research Institute, Shenzhen Key Laboratory for Nano-Biosensing Technology, School of Biomedical Engineering, Shenzhen University Medical School, Shenzhen University, Guangdong, 518060 (China).

[b] Shenzhen Key Laboratory of Marine Biotechnology and Ecology, College of Life Sciences and Oceanography, Shenzhen University, Shenzhen, Guangdong, 518055, China

[c] School of Modern Industry for Selenium Science and Engineering, Wuhan Polytechnic University, Wuhan, 430023, China

[d] Shenzhen-Hong Kong Institute of Brain Science-Shenzhen Fundamental Research Institutions, 518055, China

[e] Beijing Key Laboratory for Bioengineering and Sensing Technology, Department of Chemistry & Biological Engineering, University of Science and Technology Beijing, Beijing, 100083, China

[f] Guangdong Laboratory of Artificial Intelligence and Digital Economy (SZ), Shenzhen, Guangdong, 518107, China

[#] *These authors contributed equally to this work.*

* Corresponding authors.

Haifeng Dong

School of Biomedical Engineering, Shenzhen University Medical School,

Shenzhen University

Shenzhen, 518055, China

mail: hfdong@ustb.edu.cn; hfdong@szu.edu.cn

Qiong Liu

College of Life Sciences and Oceanography

Shenzhen University

Shenzhen, 518055, China

Tel: +86(0)75526535432

Fax: +86(0)75586713951

mail: liuqiong@szu.edu.cn

Supplementary Table 1

Antibody information.

Antibody	Host	Application	Source	Identifier
GAPDH	Rabbit	WB (1:10000)	Proteintech	Cat#10494-1-AP
GFAP	Rabbit	WB (1:1000)/<br>IF (1:200)	Proteintech	Cat#16825-1-AP
IBA1	Mouse	WB (1:1000)/<br>IF (1:200)	Proteintech	Cat#10904-1-AP
p-p38	Rabbit	WB (1:1000)	Cell Signaling	Cat#4511
p38	Rabbit	WB (1:1000)	Cell Signaling	Cat#8690
P-ERK	Rabbit	WB (1:2000)	Cell Signaling	Cat#4370
ERK	Rabbit	WB (1:1000)	Cell Signaling	Cat#4695
P-JNK	Rabbit	WB (1:1000)	Cell Signaling	Cat#4688
JNK	Rabbit	WB (1:2500)	Abcam	Cat#ab199380
TRL4	Rabbit	WB (1:1000)	Cell Signaling	Cat#14358
MyD88	Rabbit	WB (1:2000)	Proteintech	Cat#23230-1-AP
Nrf2	Rabbit	WB (1:2000)	Proteintech	Cat#16396-1-AP
HO-1	Rabbit	WB (1:10000)	Abcam	Cat#ab68477
iNOS	Rabbit	WB (1:1000)/<br>IF (1:50)	Proteintech	Cat#18985-1-AP
COX-2	Rabbit	WB (1:1000)	Proteintech	Cat#12375-1-AP
p-IκB-α	Rabbit	WB (1:1000)	Cell Signaling	Cat#2859
IκB-α	Mouse	WB (1:1000)	Cell Signaling	Cat#4814
p-p65	Rabbit	WB (1:1000)	Cell Signaling	Cat#3033
p65	Rabbit	WB (1:1000)/<br>IF (1:400)	Cell Signaling	Cat#8242

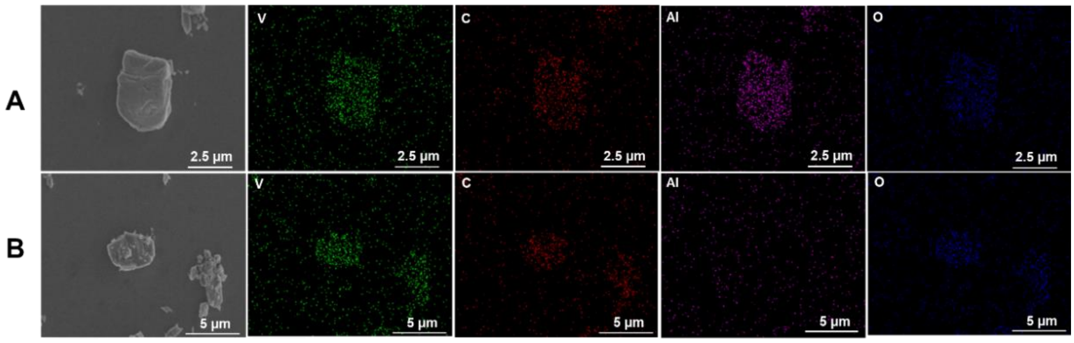

Figure S1. Energy dispersive spectroscopy (EDS) images of **(A)** V_2AlC particles and **(B)** V_2C nanosheets.

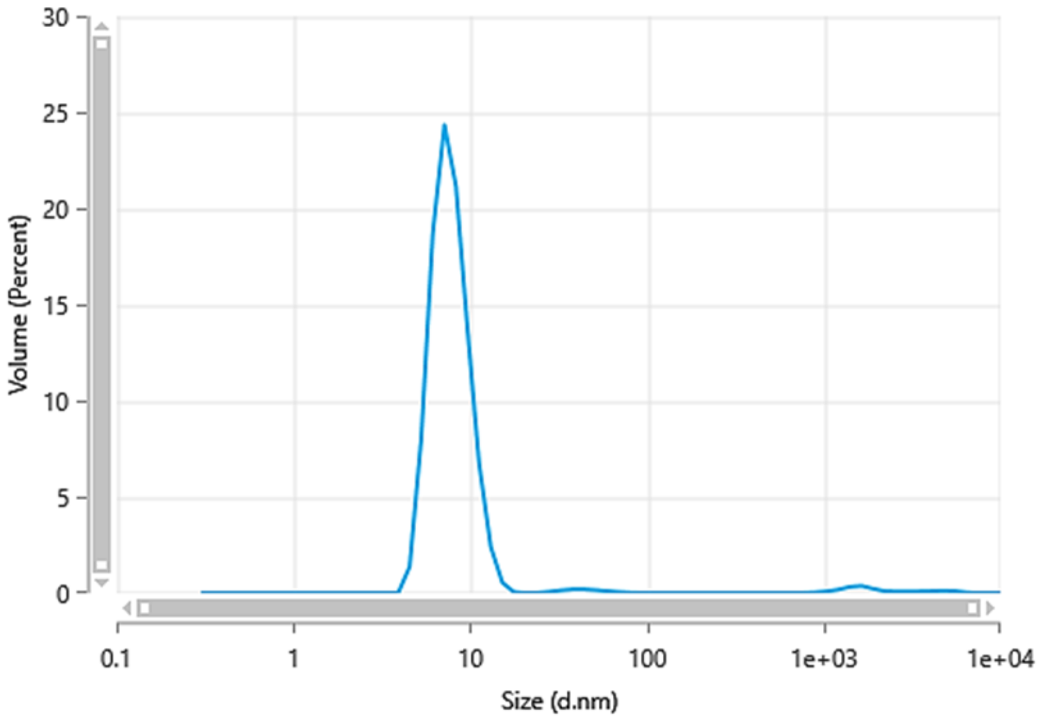

Figure S2. Size distribution of V_2C QDs measured by dynamic light scattering (DLS).

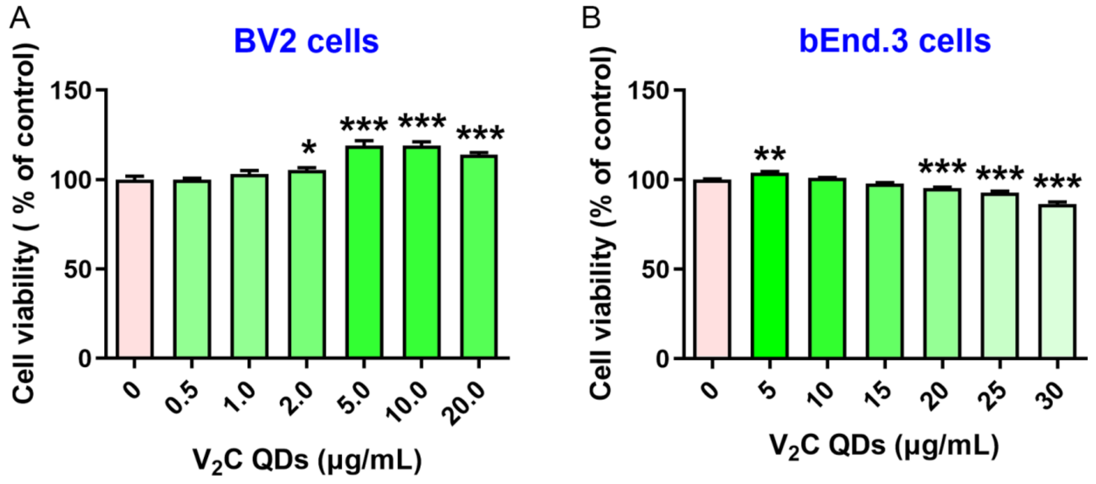

Figure S3. (A-B) CCK-8 assay of BV2 cells and bEnd.3 cells viability following treatment with different concentrations of V_2C QDs; n = 6 wells per group. $^{*}P < 0.05$, $^{**}P < 0.01$, and $^{***}P < 0.001$, V_2C QDs group vs. untreated group.

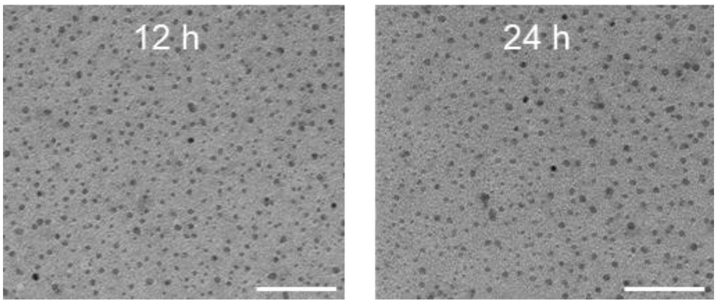

Figure S4. TEM images of V_2C QDs after cultured in the cell medium for 12 h and 24 h. (Scale bar: 50 nm)

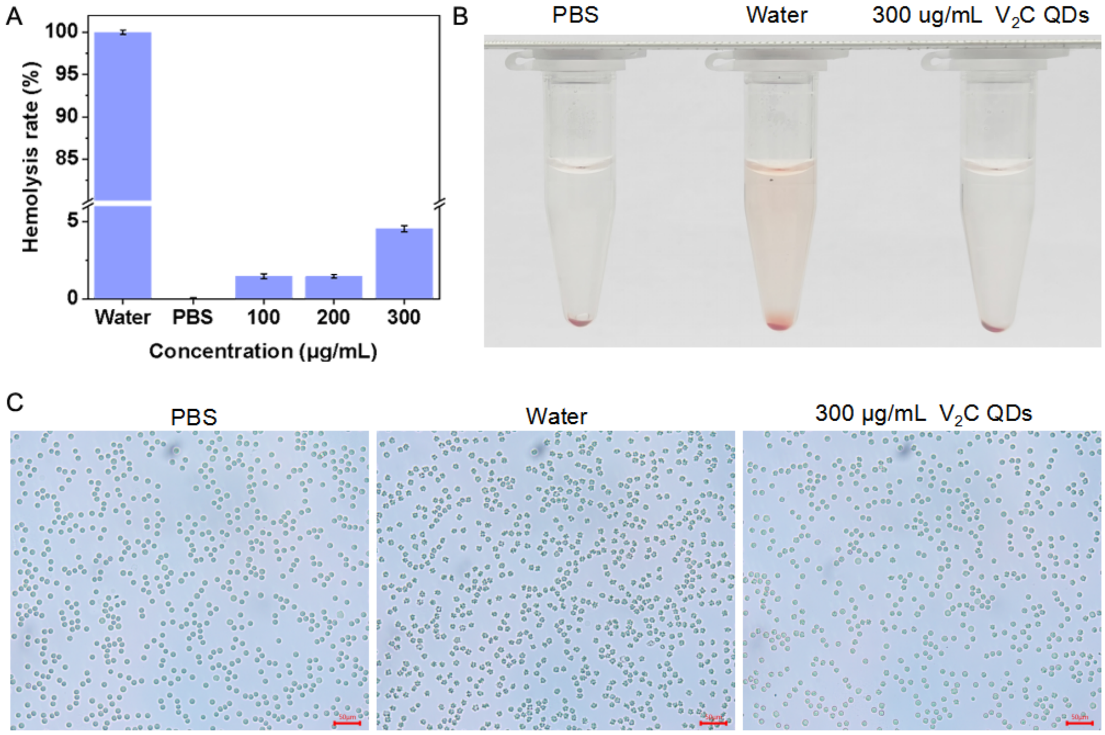

Figure S5. Biocompatibility of V_2C QDs. (**A**) Percentage of hemolysis induced by V_2C QDs. (**B**) Visual inspection of the tubes containing diluted total blood after exposure to H_2O, PBS and V_2C QDs. **(C)** Microscopic morphology of red blood cells. (Scale bar: 50 μm)

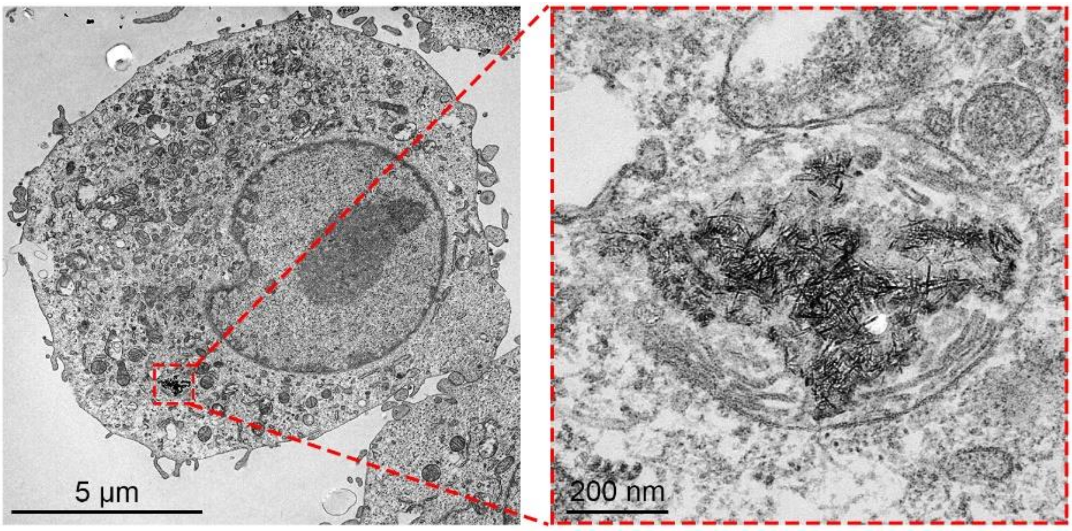

Figure S6. TEM image of V_2C QDs internalized in BV2 cells. The BV2 cells were incubated with V_2C QDs (10 μg/mL) for 8 hours.

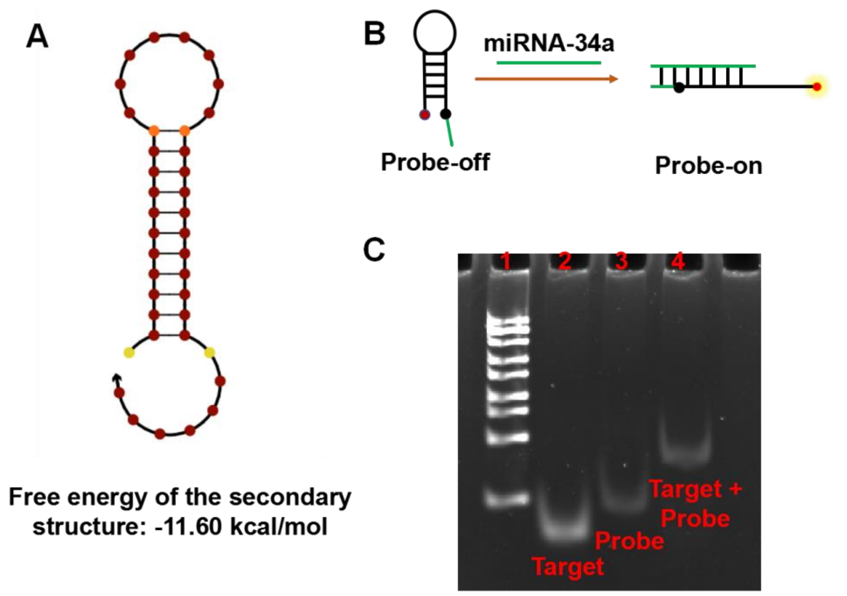

Figure S7. (A) The nucleic acid chain structure of the miRNA-34a probe was predicted using NUPACK. **(B)** Schematic illustration and **(C)** N-PAGE analysis of probe and miRNA-34a hybridization.

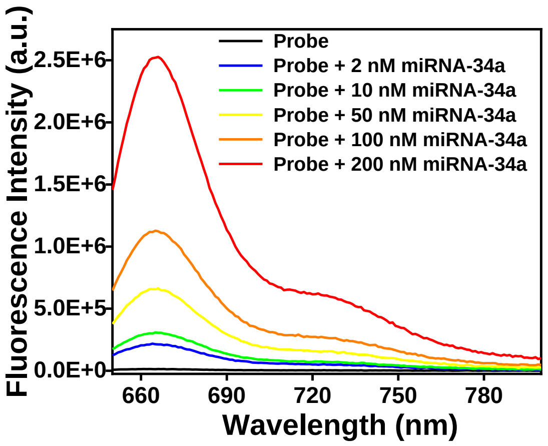

Figure S8. Fluorescence intensities of probe hybridized with different concentrations of miRNA-34a (hairpin probe, 200 nM).

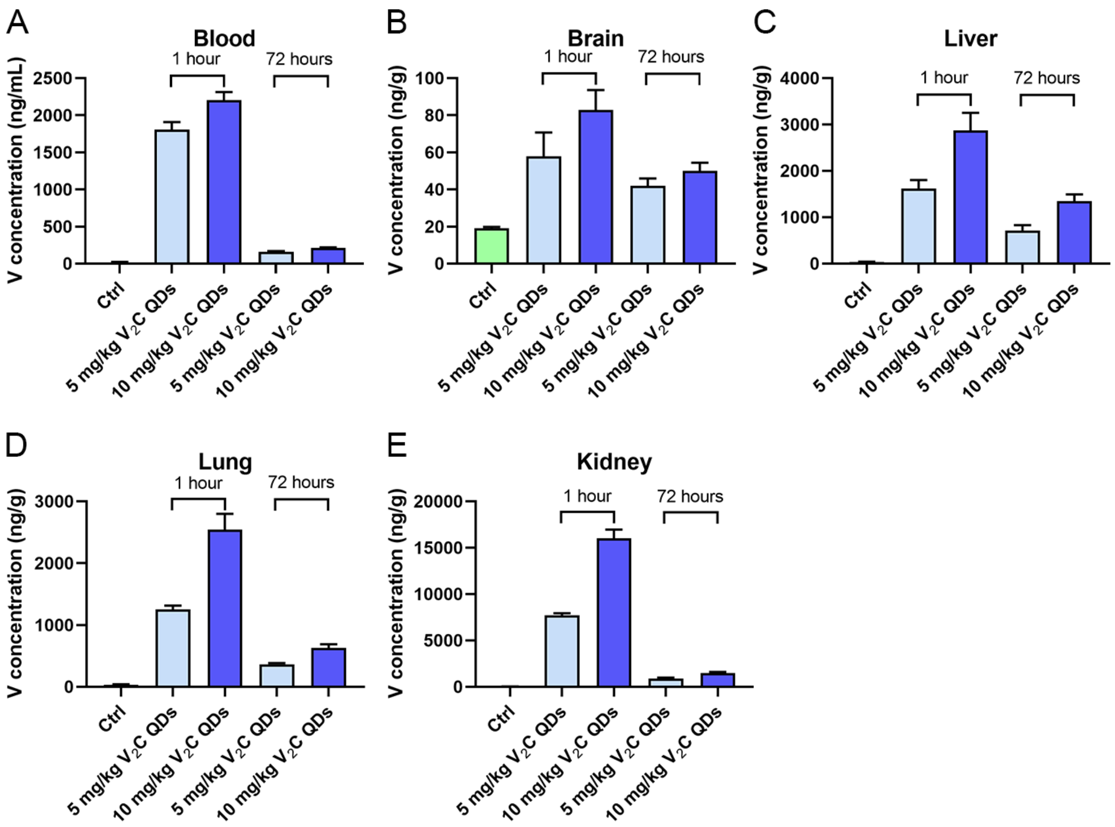

Figure S9. (A-E) Vanadium levels in the blood, brain, liver, lung and kidney of mice treated with 5 mg/kg and 10 mg/kg V_2C QDs were detected at different time points (1 h and 72 h); n = 6 mice per group.

A

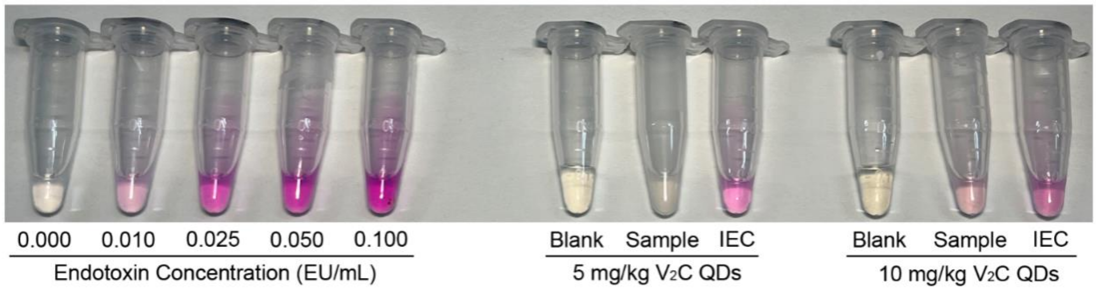

B

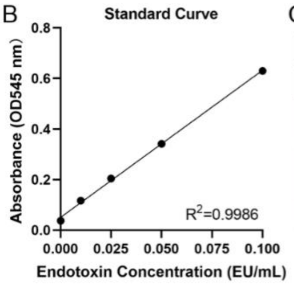

C

	5 mg/kg V_2C QDs			10 mg/kg V_2C QDs		
	Blank	Sample	IEC	Blank	Sample	IEC
Endotoxin Concentration (EU/mL)	0.000671	0.001335	0.035552	0.001003	0.011467	0.038043
Endotoxin level in injection (EU/kg mice)	0.00671	0.01335	0.35552	0.01003	0.11467	0.38043
Spike recovery (%)	-	-	68.43%	-	-	53.15%

Figure S10. (A) Image of the endpoint chromogenic LAL solution with different concentrations of endotoxin standards and the V_2C QDs samples. **(B)** Standard curve between absorbance and concentration of endotoxin standard at 545 nm. **(C)** Endotoxin concentration in different samples and endotoxin level for injection in mice. Percentages indicate percentage spike recovery. Samples spiked with endotoxin concentrations half of maximum of assay range (1/2λ_{max}: 0.050 EU/mL) and assay sensitivity (λ: 0.100 EU/mL).

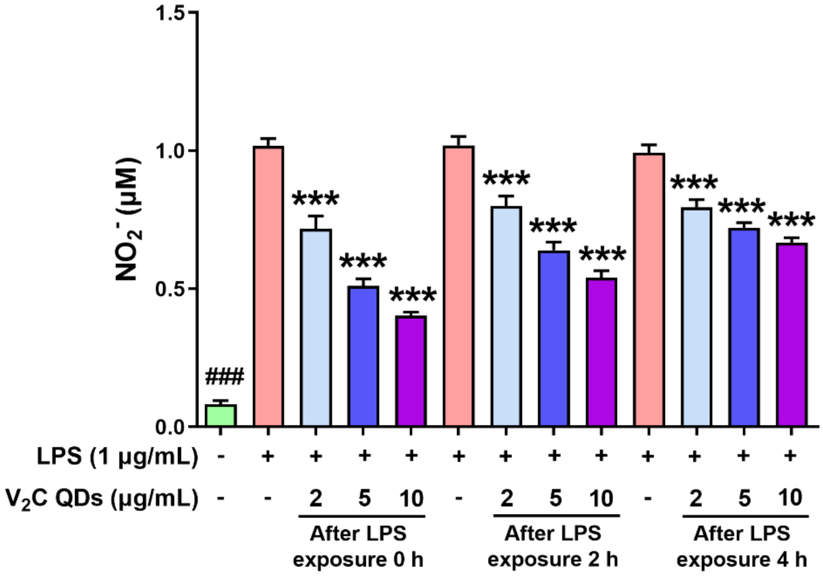

Figure S11. NO released into the culture supernatant of BV2 cells after LPS exposure at different points (0 h, 2 h and 4 h) were measured using Griess reagent.

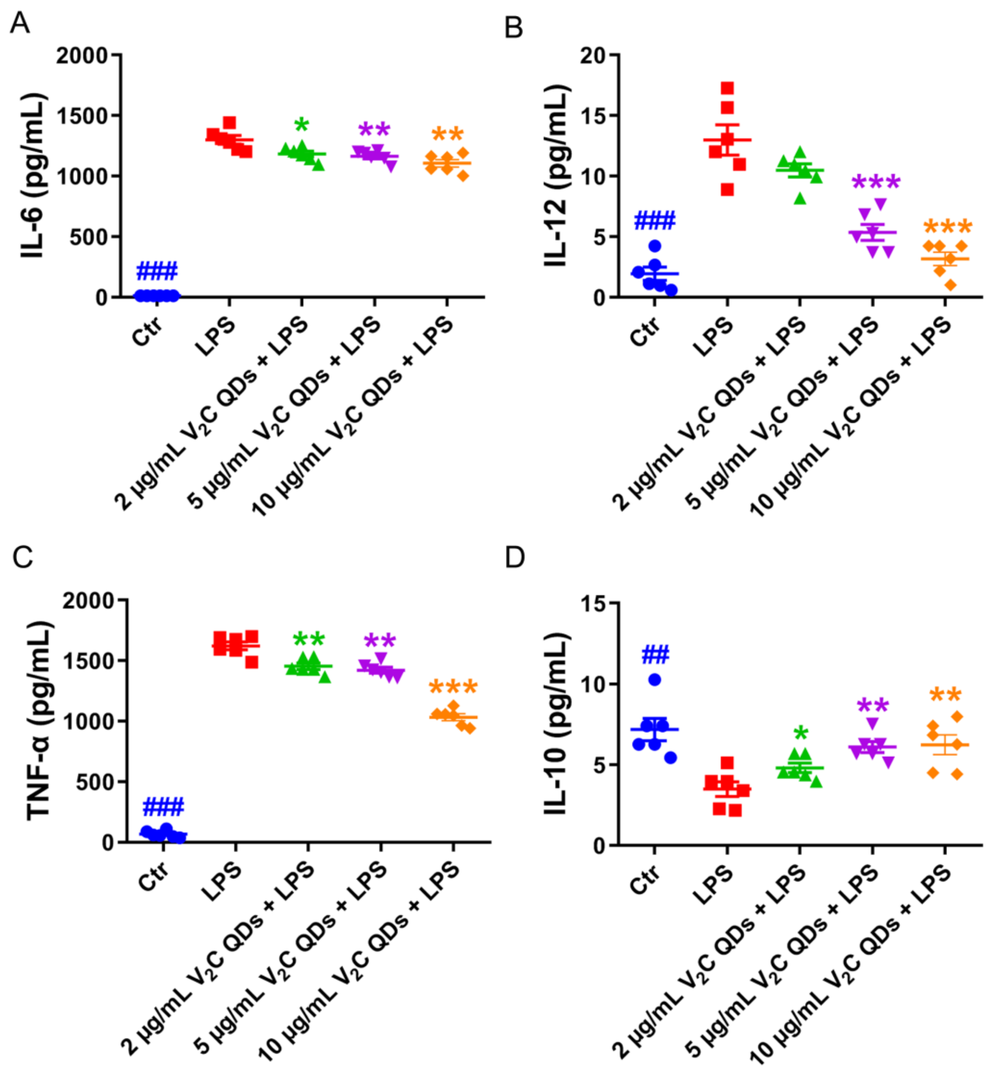

Figure S12. (A-D) Concentrations of IL-6, IL-12, TNF-α and IL-10 in culture supernatants were determined by using ELISA kits; n = 6 wells per group. $^{\#\#}P < 0.01$ and $^{\#\#\#}P<0.001$, Ctr group vs. LPS group; $^{*}P < 0.05$, $^{**}P<0.01$, and $^{***}P<0.001$, LPS + V_2C QDs group vs. LPS group.

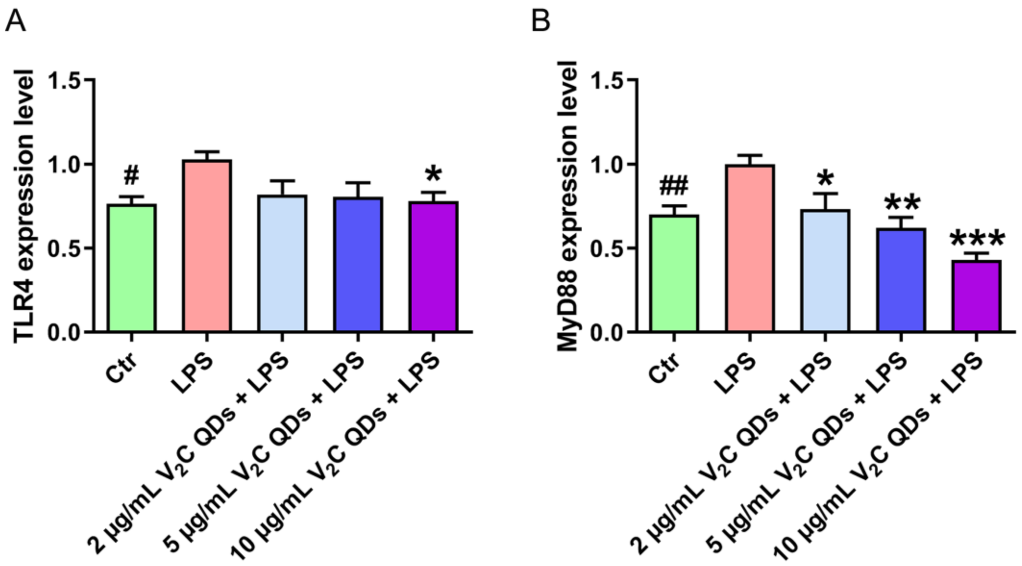

Figure S13. (**A, B**) Quantitation of TLR4 and MyD88 protein levels in BV2 cells, normalized using GAPDH; n = 3 experiments. $^{\#}P < 0.05$ and $^{\#\#}P < 0.01$, Ctr group vs. LPS group; $^{*}P < 0.05$, $^{**}P < 0.01$, and $^{***}P < 0.001$, respectively, LPS + V_2C QDs group vs. LPS group.

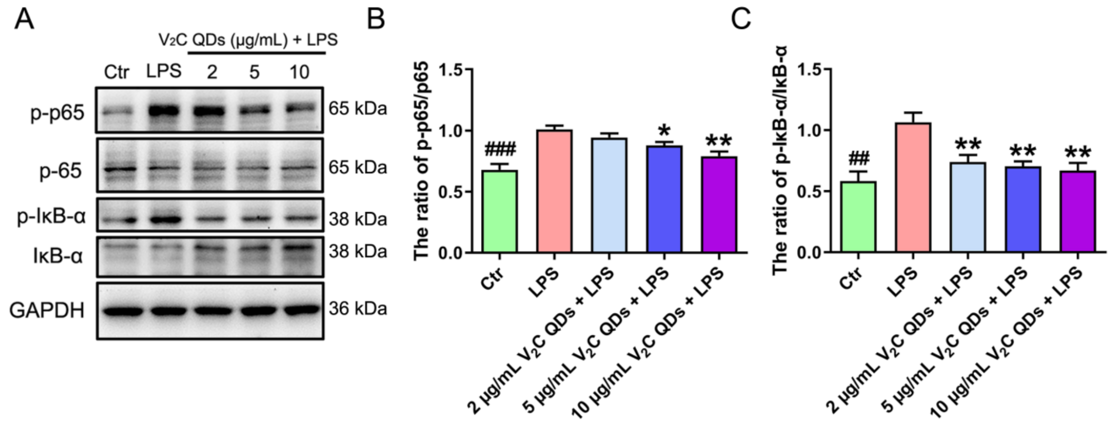

Figure S14. (A) Representative western blot analysis of p-p65, p65, p-IκB-α and IκB-α in BV2 cells. **(B-C)** Quantified and normalized ratios of p-p65/p65 and p-IκB-α/IκB-α; n = 3 experiments. $^{##}P < 0.01$ and $^{###}P < 0.001$, Ctr group vs. LPS group; $^{*}P < 0.05$, $^{**}P < 0.01$ and $^{***}P < 0.001$, LPS + V_2C QDs group vs. LPS group.

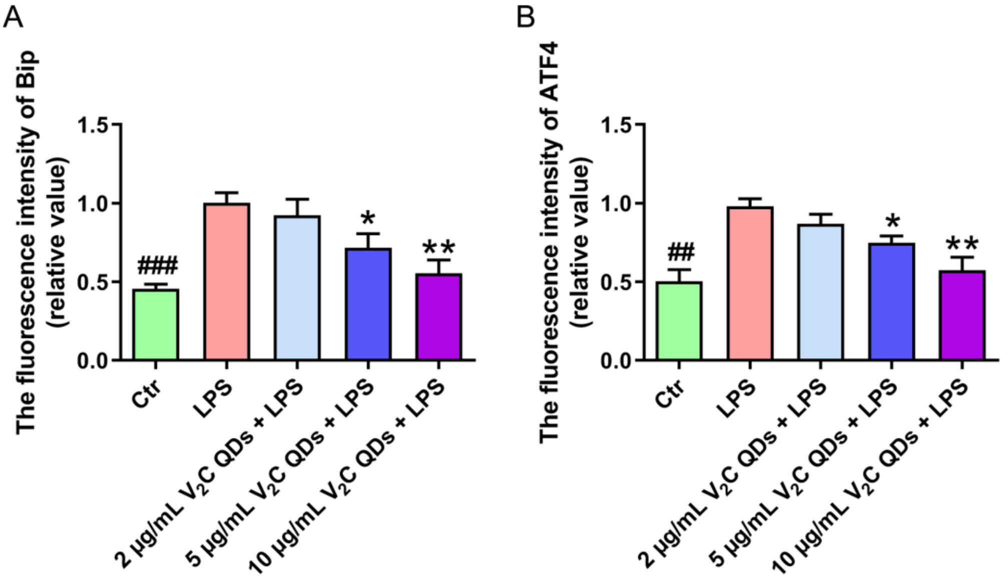

Figure S15. (**A, B**) Fluorescence intensity of Bip and ATF4 were quantified in BV2 cells using Image-Pro Plus 6.0; n = 3 experiments. $^{\#\#}P < 0.01$ and $^{\#\#\#}P<0.001$, Ctr group vs. LPS group; $^{*}P < 0.05$ and $^{**}P<0.01$, LPS + V_2C QDs group vs. LPS group.

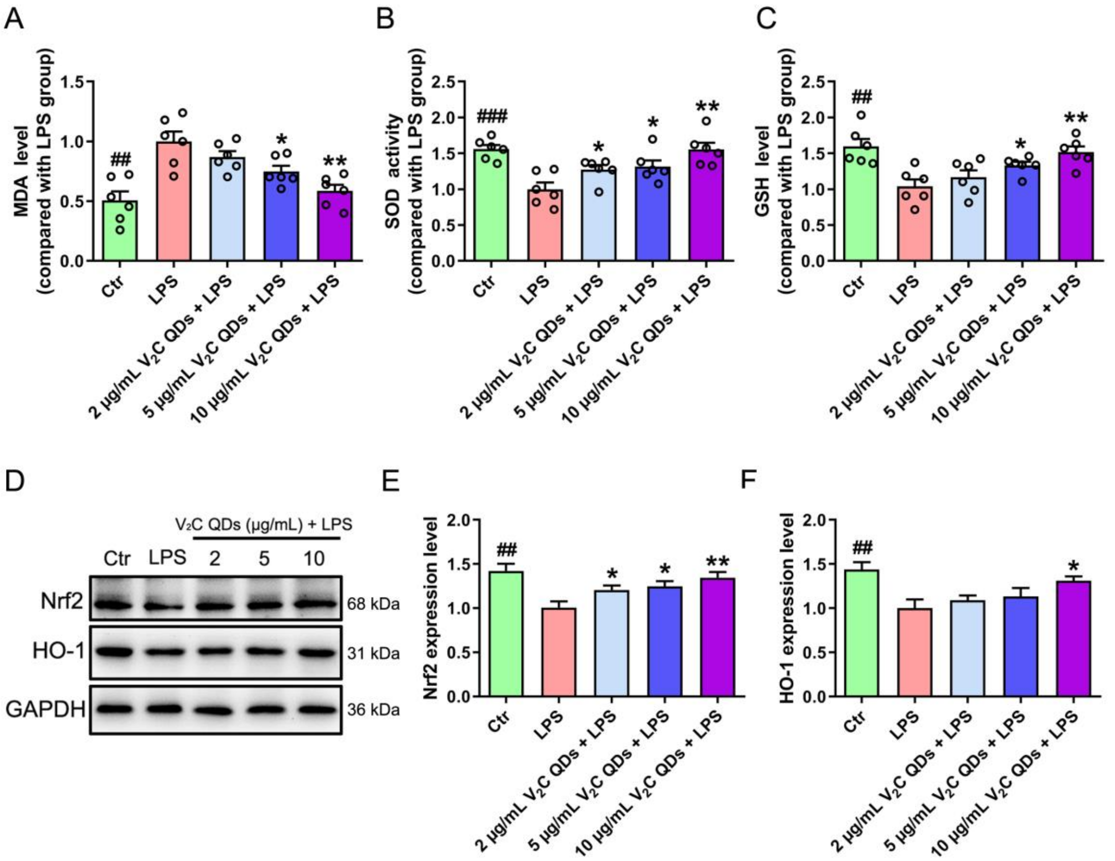

Figure S16. (A-C) SOD activity and levels of MDA and GSH in BV2 cells were measured using assay kits. **(D)** Representative western blot analysis of Nrf2 and HO-1 in BV2 cells. **(E**, **F)** Quantitation of Nrf2 (E) and (HO-1) proteins, normalized using GAPDH; n = 3 experiments; $^{\#\#}P < 0.01$ and $^{\#\#\#}P < 0.001$, Ctr group vs LPS group; $^{*}P < 0.05$ and $^{**}P<0.01$, LPS + V_2C QDs group vs. LPS group.
